# The genetics of gaits in Icelandic horses goes beyond *DMRT3*, with *RELN* and *STAU2* identified as two new candidate genes

**DOI:** 10.1186/s12711-023-00863-6

**Published:** 2023-12-11

**Authors:** Heiðrún Sigurðardóttir, Henrik Boije, Elsa Albertsdóttir, Thorvaldur Kristjansson, Marie Rhodin, Gabriella Lindgren, Susanne Eriksson

**Affiliations:** 1 Department of Animal Breeding and Genetics, Swedish University of Agricultural Sciences, P.O. Box 7023, 75007 Uppsala, Sweden; 2 Faculty of Agricultural Sciences, Agricultural University of Iceland, Borgarbyggð, 311 Hvanneyri, Iceland; 3 Department of Immunology, Genetics and Pathology, Uppsala University, Uppsala, Sweden; 4 The Icelandic Agricultural Advisory Centre, Hagatorgi 1, 107 Reykjavik, Iceland; 5 Department of Anatomy, Physiology, and Biochemistry, Swedish University of Agricultural Sciences, P.O. Box 7011, 75007 Uppsala, Sweden; 6 Department of Biosystems, Center for Animal Breeding and Genetics, KU Leuven, Kasteelpark Arenberg 30, 3001 Leuven, Belgium

## Abstract

**Background:**

In domesticated animals, many important traits are complex and regulated by a large number of genes, genetic interactions, and environmental influences. The ability of Icelandic horses to perform the gait ‘pace’ is largely influenced by a single mutation in the *DMRT3* gene, but genetic modifiers likely exist. The aim of this study was to identify novel genetic factors that influence pacing ability and quality of the gait through a genome-wide association study (GWAS) and correlate new findings to previously identified quantitative trait loci (QTL) and mutations.

**Results:**

Three hundred and seventy-two Icelandic horses were genotyped with the 670 K+ Axiom Equine Genotyping Array, of which 362 had gait scores from breeding field tests. A GWAS revealed several SNPs on *Equus caballus* chromosomes (ECA) 4, 9, and 20 that were associated (*p* < 1.0 × 10^–5^) with the breeding field test score for pace. The two novel QTL on ECA4 and 9 were located within the *RELN* and *STAU2* genes, respectively, which have previously been associated with locomotor behavior in mice. Haplotypes were identified and the most frequent one for each of these two QTL had a large favorable effect on pace score. The second most frequent haplotype for the *RELN* gene was positively correlated with scores for tölt, trot, gallop, and canter. Similarly, the second most frequent haplotype for the *STAU2* gene had favorable effects on scores for trot and gallop. Different genotype ratios of the haplotypes in the *RELN* and *STAU2* genes were also observed in groups of horses with different levels of pacing ability. Furthermore, interactions (*p* < 0.05) were detected for the QTL in the *RELN* and *STAU2* genes with the *DMRT3* gene. The novel QTL on ECA4, 9, and 20, along with the effects of the *DMRT3* variant, were estimated to account jointly for 27.4% of the phenotypic variance of the gait pace.

**Conclusions:**

Our findings provide valuable information about the genetic architecture of pace beyond the contribution of the *DMRT3* gene and indicate genetic interactions that contribute to the complexity of this trait. Further investigation is needed to fully understand the underlying genetic factors and interactions.

**Supplementary Information:**

The online version contains supplementary material available at 10.1186/s12711-023-00863-6.

## Background

Many important traits in domesticated animals are quantitative, complex traits, including performance traits in horses. Complex traits are generally regulated by a large number of genes and influenced by environmental factors. Interactions between loci and between genes, known as epistasis, may also contribute to the phenotypic variation of a trait [1, 2]. Previous studies suggest that accurate prediction of complex trait phenotypes based on genotypes requires knowledge about the existence of epistasis that may influence the trait [3, 4]. In the case of an interaction between major genes, the phenotype cannot be predicted simply by adding the effects of each locus [2, 5, 6]. In spite of an increasing number of studies on epistasis, as exemplified by the aforementioned studies, the discovery of genetic interactions on a genome-wide scale remains a major challenge [6].

In this study, we further investigated the genetic background of the economically valuable gait trait ‘pace’ in Icelandic horses. This is a complex trait that has previously been shown to be largely influenced by a single mutation in the *doublesex and mab-3 related transcription factor 3* (*DMRT3*) gene, and to be potentially influenced by epistatic effects [7].

Gait versatility is a well-known characteristic of the Icelandic horse breed. Its unique ability to perform five gaits, including the lateral gaits pace and tölt, is one of the hallmarks of the breed. Pace is a gait with a suspension phase and should ideally be ridden at high speed, making it a popular gait for racing. However, the ability to pace varies between individuals in the breed, and some individuals seem to lack it and only perform the four gaits walk, trot, canter/gallop, and tölt. Because of this, it is common to refer to Icelandic horses as being either four- or five-gaited.

The discovery that a single base change in the *DMRT3* gene influences gait variability in the Icelandic horse breed has played a key role in understanding its underlying mechanisms [7]. The ‘gait keeper’ mutation alters the pattern of locomotion and has a predominant effect on gaiting ability, in that one copy of the mutant allele [allele *A* at the *DMRT3*_Ser301STOP marker at nucleotide position 22,999,655 on *Equus caballus* chromosome (ECA) 23] enhances the natural ability to tölt, and two copies enable the development of pace [7, 8]. It has also been noted that scores from breeding field tests for the basic gaits (walk, trot, and canter/gallop) are negatively impacted by the *AA* genotype of the *DMRT3*-variant [7, 8]. In 2012, the frequency of the *A*-allele in a selected group of Icelandic horses was estimated to be 0.94 [8] and the fact that this allele is not fixed within the breed explains some of the variation in pace ability. However, there is a high proportion (> 30%) of homozygous *AA* horses that do not perform pace [7–9], and these are referred to as four-gaited in spite of their genetic precondition to develop pace. In addition, genetic variation in scores for pace among horses that did perform this gait at breeding field tests was detected in Icelandic horses [10]. Estimates of the heritability for gait scores in Icelandic horses, which are subjectively assessed by certified judges at standardized breeding field tests [11], have been reported to range from 0.18 (walk) to 0.60 (pace) [10, 12], which indicates that genetic factors as well as environmental factors influence gait quality. This is supported by the considerable genetic improvement that has been obtained in Icelandic horses during the last decades, from selection on routinely estimated breeding values for these assessed gait scores [13].

Conformation traits have been shown to discriminate between four- and five-gaited horses that are homozygous *AA* for *DMRT3* in the Icelandic horse breed to some extent [14]. Rosengren et al. [15] identified a quantitative trait locus (QTL) on ECA22 that is associated with an inclination of the backline, form of the croup, and an uphill conformation in the Icelandic horse breed. This QTL was also shown to have a considerable effect on the quality of the lateral gaits tölt and pace, which further supports the effects of conformation on gaits and highlighting the complexity of the gait traits.

Studies on other gaited horse breeds have investigated genetic differences between horses with different gait patterns but the same *DMRT3* genotype [16–18]. A pilot study of four- and five-gaited Icelandic horses with the *AA* genotype at the *DMRT3* gene did not reach conclusive results due to a rather small number of horses (20 four-gaited and 35 five-gaited horses) [19]. Thus, knowledge about the genetic background of gaiting ability and the quality of gaits in Icelandic horses is still incomplete. The aim of the present study was to further investigate the genetic background of pace in Icelandic horses, using a larger dataset to identify novel genetic factors that influence pacing ability and quality through a genome-wide association study (GWAS). A second aim was to investigate how the new identified QTL interacted with previously reported QTL and mutations. This is a first step towards understanding the complex genetic background that underlies the phenotype of pace beyond the *DMRT3* gene, or possibly interacting with it.

## Methods

### Animals

The study included 372 privately owned Icelandic horses born between 1993 and 2016, among which 160 were stallions or geldings, and 212 were mares. All horses had been shown at a breeding field test, except for 10 geldings that were specialized in pace racing. Hair samples from some of the horses were originally collected for an unrelated study with selection of horses based on the mane and tail characteristics. The remaining horses were selected from breeding field tests in 2020 with inclusion of both individuals with high and low assessment scores from the tests. Hair samples were collected from the horses’ tails, with informed consent from the owners. Collection was done at breeding field tests and during visits to trainers and breeders in Iceland and Sweden. Pedigree data were obtained from the international Worldfengur database [20]. The maximum relatedness between individuals in the sample was a half-sib relationship, and efforts were made to balance the contributions from different families and avoid stratification in the data. The number of unique sires and dams for the individuals in the study was 244 and 362, respectively.

### Phenotype data

The phenotype data used were retrieved from the Worldfengur database [20] and consisted of scores for pace recorded at standardized breeding field tests for Icelandic horses between 1999 and 2022 [11]. Scores for other gaits assessed at breeding field tests were also used to further investigate the effects of the genomic regions that were found to be associated with pace. In cases where horses had been assessed at more than one breeding field test, the highest assessment score was used. Most horses were assessed in Iceland (N = 269) and the others were assessed in Denmark (N = 1), Germany (N = 6), Norway (N = 1), and Sweden (N = 85). The age of the horses when they attended a breeding field test ranged from 4 to 15 years (mean = 6.7 years).

### Pace

Pace is described as a symmetrical, two-beat gait in which ipsilateral legs move nearly synchronously back and forth with a brief moment of suspension. Pace is an energetic gait ridden at high speed, where the horse lengthens its strides, lifts its back, and extends the head and neck forward. Pure pace is characterized by a visible suspension phase where none of the hoofs touch the ground and divergence from synchronous movements of the ipsilateral legs is not noticeable [21].

The quality of pace is subjectively assessed by a panel of certified judges at standardized breeding field tests and scored on a scale from 5 to 10 with 0.5 intervals [11]. To receive the highest score for pace, the horse should be able to pace steadily in good balance, with long strides, elegant and light movements, good suspension, and excellent speed. Correct body function and a long, strong topline where the horse extends its head and neck forward are of great importance for the higher scores. A faulty and/or uneven beat in pace, lack of speed, stiff movements, short strides, and a concave topline are considered poor qualities of pace and entail lower scores. A score of 5 is given only if the horse does not present the pace trait [11].

**Fig. 1:**
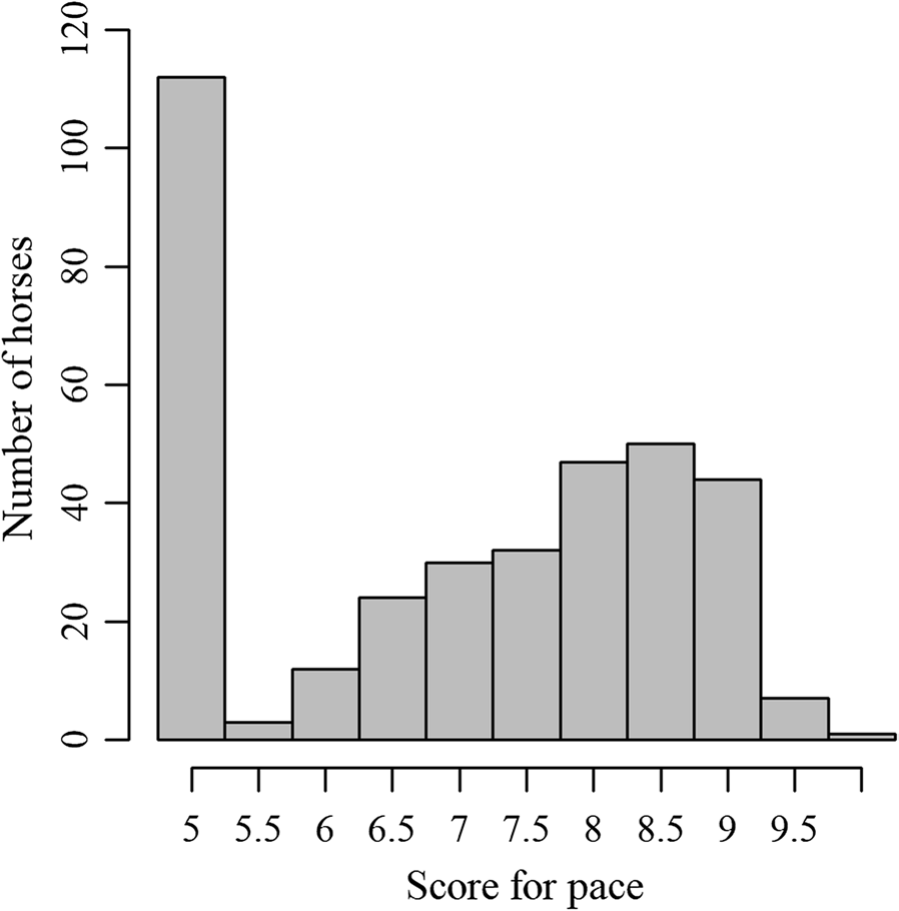
Distribution of scores for pace. Distribution of scores for pace in the sample of 362 horses assessed at a breeding field test

The scores for pace that the horses received in this study ranged from 5.0 to 10.0, with a mean of 7.0 [standard deviation (SD) 1.55]. The distribution of the scores for pace higher than 5.0 was slightly negatively skewed (− 0.13) (Fig. 1). Transformation of the data to increase normality was tested but did not affect the results. The distributions after rank- and log transformation of the pace scores are presented in Additional file 1: Fig. S1.

### Other traits assessed at breeding field tests

In addition to pace, scores for the gait traits tölt, trot, canter, gallop, and walk were used to investigate the effects of the detected genomic regions associated with pace. As for pace, the quality of these gaits was subjectively assessed by the judging panel and scored on a scale from 5 to 10 with 0.5 intervals [11]. Tölt is a symmetrical four-beat ambling gait with an ipsilateral sequence of footfall and a large speed variation but without a suspension phase. Trot is a symmetrical two-beat, diagonal gait with a moment of suspension. Both canter and gallop are asymmetrical gaits with a suspension phase but canter is characterized as a three-beat, medium-speed gait, whereas gallop is defined as a four-beat, high-speed gait. Walk is a symmetrical, four-beat stepping gait with an ipsilateral movement and without suspension [21].

Descriptive statistics and distributions of the scores for these traits are in Additional file 2: Table S1 and Additional file 3: Fig. S2. The distribution of these scores did not differ significantly from a normal distribution based on a Jarque–Bera normality test (*p* > 0.05), except the distribution of scores for tölt. However, transformation of the data to increase normality did not affect the results. The distributions after rank- and log transformation of the scores for tölt are in Additional file 4: Fig. S3.

**Fig. 2:**
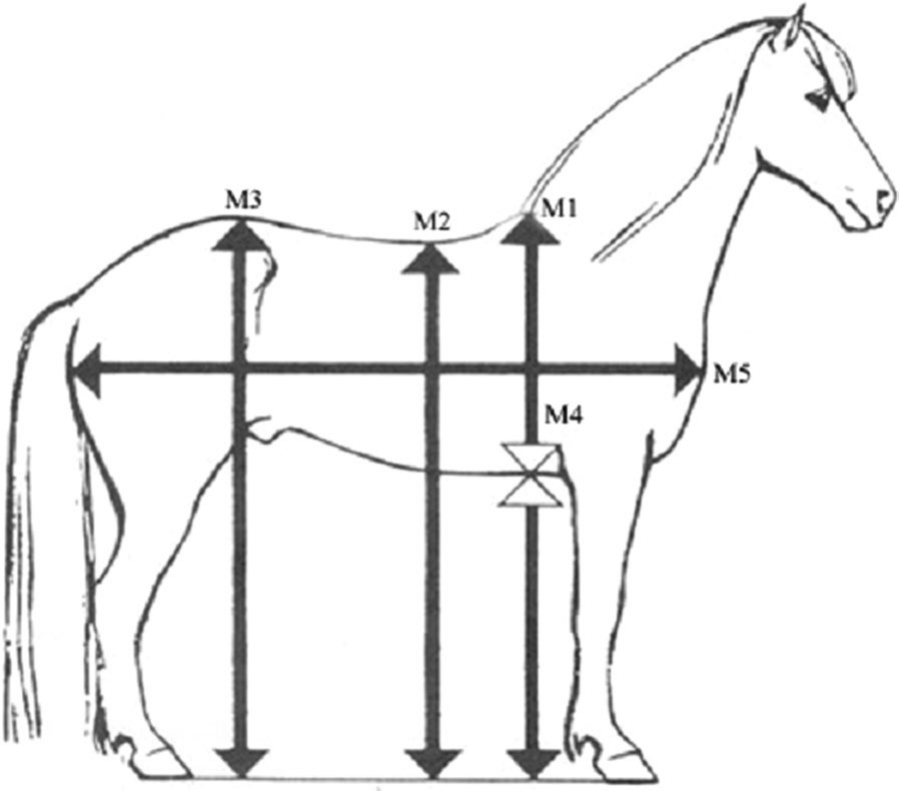
Morphological measurements recorded at standardized breeding field tests for Icelandic horses [22]. Original image created by Pétur Behrens

Morphological measurements are also recorded at breeding field tests [22]. These measurements, made with a rod, include height at withers (M1), height at the lowest point of the back (M2), height at croup (M3), and depth of breast (M4) (Fig. 2), among others. These measurements are used to assess the longitudinal balance of the horse in standstill position and its level of uphill conformation by comparing the height of the front to that of the hind part (M1-M3) and by calculating height of the withers (M1-M2).

### DNA preparation, genotyping, and quality control

DNA was extracted from approximately 25 hair roots from each horse, which were digested in 200 µl of 5% Chelex 100 Resin (Bio-Rad Laboratories, Hercules, CA, US) and 14 µl of proteinase K (20 mg/ml; Merck KgaA, Darmstadt, Germany) by incubation for 2 h at 56 °C at 900 rpm, followed by heat inactivation of the proteinase K at 95 °C for 10 min. After centrifugation, 150 µl from the top of each sample were withdrawn, leaving the Chelex and hair roots in the tube; to obtain more DNA, some of the samples were submitted to a second round of digestion, i.e. 100 µl of 5% Chelex 100 Resin and 7 µl of proteinase K were added and the sample were incubated again for 2 h at 56 °C. DNA concentration was determined using a NanoDrop-2000 (Thermo Fisher Scientific, Wilmington, DE, USA) and a Qubit 3.0 fluorometer (Life Technologies).

The 372 DNA samples were genotyped with the 670 K+ Axiom Equine Genotyping Array. Quality control (QC) was performed using the GenABEL package [23, 24] in R (v.3.6.1) [25]. Poorly genotyped samples and noisy data were removed based on the following criteria: if the rate of missing genotypes per single nucleotide polymorphism (SNP) was higher than 0.10, the rate of missing SNPs per sample was higher than 0.10, the minor allele frequency (MAF) was lower than 0.05, and if there was deviation from Hardy-Weinberg equilibrium (*p*-value ≤ 1.0 × 10^−10^). Genotyping of the *DMRT3*_Ser301STOP SNP, known as the ‘gait keeper’ mutation [7], was performed manually on 25 samples that yielded a low call rate for this marker in the 670 K+ Axiom Equine Array genotyping, using custom-designed TaqMan SNP Genotyping Assays (Applied Biosystem). The 10-µl reaction volume contained: 1 µl DNA (concentration 150–170 ng/µl), 0.25 µl Genotyping Assay 40X, 5 µl Genotyping Master Mix 2X, and 3.75 µl deionized water and the following PCR conditions: 95 °C for 10 min, 40 cycles of 95 °C for 15 s, and 60 °C for 1 min. Results revealed that most of the sampled horses, i.e. 340, carried two copies of the mutant allele (*AA* genotype), 31 individuals carried one copy (*CA* genotype), and one individual carried none (*CC* genotype).

### Genome-wide association study

A multidimensional scaling (MDS) plot was constructed based on a genomic-kinship matrix obtained with the ibs() function in the GenABEL package [23, 24] to identify potential stratification among the individuals. Different fixed effects were tested with the lm() function for a linear model in R (v.3.6.1) [25] using analysis of variance (ANOVA) as a post-hoc test, including sex (male or female), *DMRT3* genotype, age at assessment (4, 5, 6, and ≥ 7 years old), age in years at assessment as a covariate, country of assessment (Iceland versus other countries), and year of assessment (≤ 2016, 2017–2019, 2020, and 2021–2023). None of these effects were significant (*p* ≤ 0.05) for the score for pace, except the effects of sex and of *DMRT3* genotype, and these were included in further analyses.

The GenABEL package [23, 24] in R (v.3.6.1) [25] was used to perform the GWAS for pace score. The genomic-kinship matrix obtained with the ibs() function, together with sex and *DMRT3* genotype as fixed effects, were used in a hierarchical generalized linear model for pace score with the polygenic_hglm() function [26]. This was done to estimate residuals and the inverse of the variance-covariance matrix, to be subsequently used in a mixed model-structured association approach with the mmscore() function in the GenABEL package [23, 24]. Genome-wide significance was determined by Bonferroni correction (*p* < 6.9 × 10^−8^), with a suggestive genome-wide significance threshold set at 1.0 × 10^−5^ [27, 28].

An additional analysis was done using only pace scores for horses with the *AA* genotype for *DMRT3*. This did not alter the general results in terms of the detected genomic regions and, therefore, to retain more observations in the analysis, we chose to include all *DMRT3* genotypes and account for the fixed effects of *DMRT3* genotype in the GWAS.

### Functional annotation of candidate genes

The genome browser Ensembl (release 108, Oct 2022) [29] was used to screen for candidate genes based on the EquCab3.0 reference genome and QTL annotation in the HorseQTLdb (release 49, Dec 2022) [30] was used to search for known QTL for gait and performance traits in horses. Functional annotation of possible candidate genes was performed using the GeneCards database (version 5.14, Jan 2023) [31, 32]. All positions refer to the EquCab3.0 reference genome.

### Haplotype analyses

To visualize linkage disequilibrium (LD) between the significant SNPs from the GWAS, an LD Manhattan plot was constructed using the package cgmisc 2.0 [33] in R (v.3.6.1) [25]. Calculations for pairwise LD were also performed in PLINK (v.1.9) [34, 35] using the --r2 command. A haplotype analysis was performed with the haplo.stats package [36] in R (v.3.6.1) [25]. The function haplo.em() was used to estimate the frequency of the different haplotypes derived from significant SNPs that were in significant LD (r^2^ ≥ 0.8). The haplotype effect on the pace score was estimated using a generalized linear model with the function haplo.glm(). The most frequent haplotype was used as a reference and only haplotypes with frequencies higher than 0.02 were included. An empirical *p*-value was estimated by using 100,000 permutations based on an additive effect.

Phenotypic variation of the scores for pace was analyzed with the polygenic_hglm() function in the GenABEL package [23, 24] in R (v.3.6.1) [25] to quantify the variation explained by significant haplotypes and top SNPs. A phenotype association analysis of the horses that were homozygous for the haplotypes that had a significant effect on pace was conducted using a one-way ANOVA based on a general linear model procedure in R (v.3.6.1) [25]. The significance level was set at a *p*-value ≤ 0.05. All gaits assessed at breeding field tests were tested. Gait scores were corrected for the fixed effects of sex and *DMRT3* genotype.

The horses were divided into five groups based on their ability to pace and the haplotype frequency within each group was investigated. The groups comprised (1) all horses with the *AA* genotype at the *DMRT3*-variant (*AA* horses, N = 340), (2) horses with the *AA* genotype at the *DMRT3*-variant and that had shown pace at a breeding field test (5-gaited *AA* horses, N = 248), (3) horses with the *AA* genotype at the *DMRT3*-variant and that had been shown at a breeding field test but had not shown pace during the test (4-gaited *AA* horses, N = 82), (4) horses with the *CA* genotype at the *DMRT3*-variant (*CA* horses, N = 31), and (5) horses with the *AA* genotype at the *DMRT3*-variant that had raced in a pace competition but had not attended a breeding field test (pace racers, N = 10). In addition, the whole sample was entered as a reference group, including the single horse with the *CC* genotype at the *DMRT3*-variant (All horses, N = 372). Significance of differences in genotype frequencies between the groups was estimated with the pairwise_prop_test() function in the rstatix package [37] in R (v.3.6.1) [25], a post-hoc test following a significant chi-square test of homogeneity.

Interactions between the detected significant haplotypes and the ‘gait keeper’ mutation in the *DMRT3* gene [7], as well as the previously identified QTL on ECA22 associated with the conformation of the back and croup [15], were investigated for all gaits. Interaction effects were investigated by constructing boxplots with the ggplot2 package [38] in R (v.3.6.1) [25], using ANOVA and Tukey’s test as a post-hoc test. The significance level was set at *p*-value ≤ 0.05.

## Results

### Genotyping and quality control

**Fig. 3:**
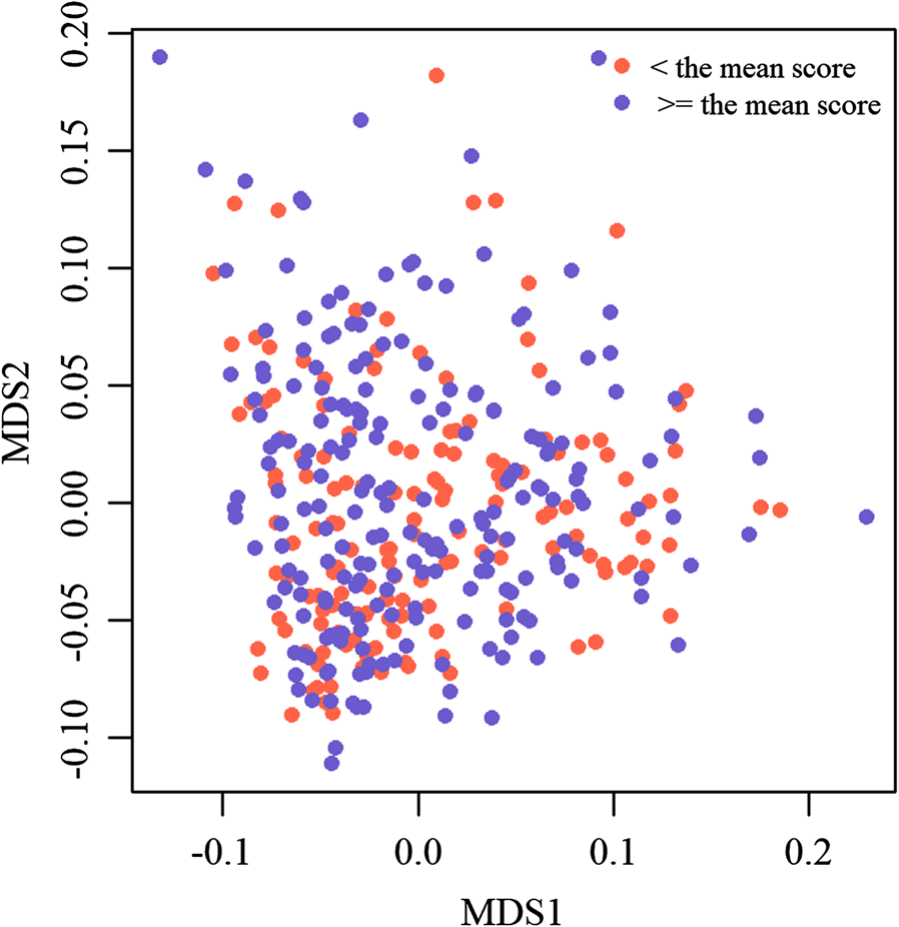
MDS plot for pace score. Visualization of population stratification across the horses that passed quality control and had a record for the phenotype. Red represents horses that had a score lower than the mean 7.0 and blue represents horses with a score higher or equal to 7.0

In total, 361,333 SNPs (358,655 on autosomes and 2678 on the X chromosome) and 372 horses passed QC and were included in the GWAS. One horse was excluded from the dataset because of too high identity-by-state (IBS) with another horse, i.e. greater than 0.95. No apparent outliers or stratification in the data was detected in the MDS plot (Fig. 3).

### Genome-wide association analysis

**Fig. 4:**
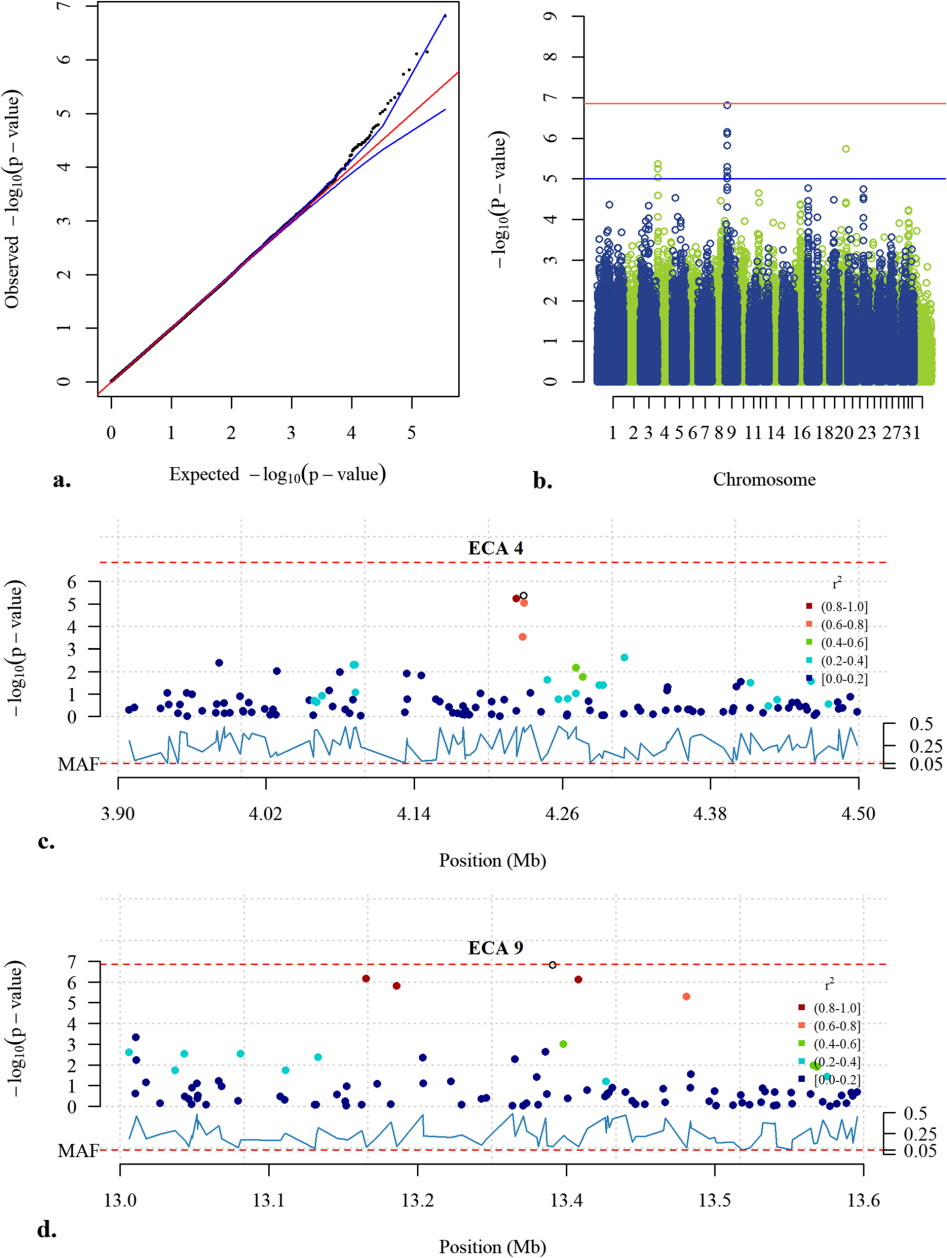
GWA results for the score for pace. **a** Quantile-quantile (QQ) plot where the blue lines represent the 0.05–0.95 confidence interval. The estimated lambda value was 0.99 (se 2.84 × 10^−5^). **b** Manhattan plot from the mixed model association analysis. The red horizontal line indicates the Bonferroni significance threshold (*p* < 6.9 × 10^−8^) and the blue horizontal line indicates the suggestive genome-wide significance level (*p* < 1.0 × 10^−5^). **c** LD Manhattan plot on ECA4 with the top SNP as an open circle. Three SNPs reached the suggestive threshold of which two were in LD (r^2^ ≥ 0.8). **d** LD Manhattan plot on ECA9 with the top SNP as an open circle. Seven SNPs reached the suggestive threshold of which four were in LD (r^2^ ≥ 0.8). All positions refer to the EquCab3.0 genome assembly

Three SNPs, located on ECA4 between 4,222,615 and 4,228,914 bp, reached the suggestive threshold (*p* < 1.0 × 10^–5^) and two of them had high LD (r^2^ ≥ 0.8) (Fig. 4b, c). Seven SNPs on ECA9 between 11,533,922 and 13,457,268 bp also reached the suggestive threshold, of which one almost reached the Bonferroni significance threshold (*p* < 6.9 × 10^−8^) (Fig. 4b). Four of the top SNPs on ECA9 were in LD (Fig. 4d). One SNP reached the suggestive threshold on ECA20 at 52,057,378 bp, and was not in LD with any other SNP in the region (Fig. 4b). A summary of the GWA results for the 50 top SNPs is presented in Additional file 5: Table S2.

### Candidate genes related to pace

The region detected on ECA4: 4,222,615–4,228,914 bp was located within the *reelin* (*RELN*) gene and the locations of the SNPs relative to the *RELN* gene’s exon and intron positions are presented in Additional file 6: Fig. S4). Ten other genes were found in the vicinity of the detected region on ECA4 (see Additional file 7: Table S3). Regarding the region detected on ECA9: 11,533,922–13,457,268 bp, the five SNPs with the lowest *p*-value within this region were located within or in near proximity (~ 4 kb) of the annotated *staufen double-stranded RNA binding protein 2* (*STAU2*) gene; their locations relative to the gene’s exon and intron positions are presented in Additional file 8: Fig. S5. Nine other genes were found in the vicinity of this region (see Additional file 7: Table S3). The region located on ECA20: 52,057,378 bp contained a single SNP that was located within a long non-coding RNA gene (*ENSECAG00000046047*) and 12 protein-coding genes and 12 other long non-coding RNA genes were found in the vicinity of this region (see Additional file 7: Table S3). None of the suggestive SNPs (on ECA4, 9, and 20) overlapped with any known QTL for gait traits in horses [30].

### Haplotype analyses

Haplotype analysis for the identified region on ECA4 revealed two haplotypes that resulted in higher and lower scores for pace, respectively (*p*-value < 0.001) (Table 1). Ninety-nine horses were homozygous for the haplotype associated with a higher score for pace (R haplotype) and 65 horses were homozygous for the haplotype associated with a lower score for pace (r haplotype). The pooled group of rare haplotypes (haplotype frequency < 0.02) had a frequency of 0.01 (results not presented).

The haplotype analysis for the region identified on ECA9 also revealed two haplotypes which resulted in higher and lower scores for pace, respectively (*p*-value < 0.001) (Table 1). In total, 298 horses were homozygous for the haplotype associated with a higher score for pace (S haplotype) and five horses were homozygous for the haplotype associated with a lower score for pace (s haplotype). The pooled group of rare haplotypes had a frequency of 0.02 (results not presented).

No haplotype analysis was performed for the SNP identified on ECA20 since it was not in LD with any other SNP in the region. The allele frequencies of this SNP were 0.90 for allele *A* and 0.10 for allele *C*.

**Table 1:** Results from the haplotype analysis for SNPs on ECA4 and 9 associated with pace score

Chr	Hap. ID	Haplotypes (SNP number^a^)				Freq	Coeff	*p*-value	Sim. *p*-value	${{\sigma }}_{{p}}^{2}$ ratio
ECA4		1		2^b^						
	R	*C*		*C*		0.54	0.51	< 0.001	< 0.001	0.044
	r	*A*		*A*		0.45	− 0.51	< 0.001	< 0.001	
ECA9		1	2	3^c^	4					
	S	*A*	*A*	*A*	*G*	0.90	0.89	< 0.001	< 0.001	0.044
	s	*G*	*C*	*G*	*A*	0.08	− 0.89	< 0.001	< 0.001	

Chr: chromosome; Hap. ID: haplotype identification letters to distinguish between different haplotypes; Freq: haplotype frequency; Coeff: coefficient, estimated effect of the haplotype on the pace score from a GLM regression in the haplotype analysis; Sim. *p*-value: *p*-value adjusted by using 100,000 permutations; ${\sigma }_{p}^{2}$ ratio: ratio of phenotypic variance explained by the haplotypes

^a^SNP numbers are in bp positional order

^b^The top SNP on ECA4 is number 2 in the positional order, where the reference allele is *A* and alternate allele is *C*

^c^The top SNP on ECA9 is number 3 in the positional order, where the reference allele is *G* and alternate allele is *A*

The proportion of phenotypic variation of the pace score explained by the significant haplotypes in the *RELN* and *STAU2* genes was 4.4% for each (Table 1). The proportion of phenotypic variation explained by the top SNP on ECA20 was 4.2%. The effects of *DMRT3* genotype combined with the effects of the significant haplotypes of the *RELN* and *STAU2* genes and the top SNP on ECA20 accounted for 27.4% of the phenotypic variance of the pace score. The *DMRT3*-variant explained 13.7% of the phenotypic variance.

ANOVA revealed that the group of individuals that were homozygous for the R haplotype (RR genotype) differed significantly in mean scores (*p*-value ≤ 0.05) from those that were homozygous for the r haplotype (rr genotype) for all the gait phenotypes assessed at breeding field tests, except for walk (Table 2). These two groups differed significantly for the morphological measurements of height of the withers (from the lowest point on the back to the highest point on the withers) and of height at the front compared to the hind part (height at the croup to height at the withers). While horses with the RR genotype had a higher mean score for pace, horses with the rr genotype had higher mean scores for all the other gaits, height of the withers, and height at the front compared to the hind part (more uphill conformation).

**Table 2:** Results from the ANOVA that compared phenotypes in horses with different QTL genotypes in the *RELN* gene

	ECA4-RR			ECA4-rr			*t*-value	*p*-value	df
	N	LSmeans	se	N	LSmeans	se			
Gait traits^a^									
Tölt	99	8.30	± 0.07	65	8.62	± 0.10	3.39	0.001	356
Slow tölt	97	8.06	± 0.07	64	8.39	± 0.10	3.25	0.001	351
Trot	99	7.95	± 0.07	65	8.34	± 0.10	3.83	< 0.001	356
Pace	99	7.45	± 0.16	65	6.46	± 0.22	− 4.49	< 0.001	356
Gallop	99	7.99	± 0.06	65	8.27	± 0.08	3.33	0.001	356
Canter	97	7.66	± 0.06	64	8.08	± 0.09	4.62	< 0.001	350
Walk	99	7.79	± 0.07	65	7.87	± 0.08	0.75	0.455	356
Morphological traits^b^									
Height of withers (M1–M2)	90	10.21	± 0.19	63	10.82	± 0.26	2.32	0.021	334
Height at front (M1–M3)	98	4.27	± 0.21	64	4.90	± 0.30	2.06	0.040	353

*N* number of horses, *LSmeans* least squares means, *se* standard error, *df* degrees of freedom

^a^Subjectively assessed traits (judging scale 5–10)

^b^Morphological measurements (cm)

Due to the high frequency of homozygotes for the S haplotype (SS genotype) at the *STAU2* gene and the low frequency of homozygotes for the s haplotype (ss genotype), the ANOVA yielded no significant results when comparing the means for these two groups. However, a comparison of horses with the SS genotype with the rest of the sample, including horses with the ss or Ss genotype, and horses carrying other rare haplotypes (2% of the sample) revealed significant differences (*p*-value ≤ 0.05) for pace, trot, and gallop (Table 3). The horses with the SS genotype had a higher mean score for pace but lower mean scores for all the other gaits, compared to all other horses. Morphological measurements were not significantly different (*p*-value > 0.05) between these two groups of horses.

**Table 3:** Results from the ANOVA that compared phenotypes in horses with different QTL genotypes in the *STAU2* gene

Gait traits^a^	ECA9-SS			ECA9-Ss + ss			*t*-value	*p*-value	df
	N	LSmeans	se	N	LSmeans	se			
Tölt	298	8.35	± 0.05	64	8.49	± 0.08	1.70	0.091	357
Slow tölt	295	8.14	± 0.05	62	8.24	± 0.09	1.11	0.268	352
Trot	298	8.05	± 0.05	64	8.28	± 0.09	2.62	0.009	357
Pace	298	7.15	± 0.10	64	6.13	± 0.19	-5.39	< 0.001	357
Gallop	298	8.08	± 0.04	64	8.29	± 0.07	3.01	0.003	357
Canter	294	7.82	± 0.04	62	7.97	± 0.08	1.93	0.054	351
Walk	298	7.81	± 0.05	64	7.84	± 0.09	0.29	0.770	357

*N* number of horses, *LSmeans* least squares means, *se* standard error, *df* degrees of freedom

^a^Subjectively assessed traits (judging scale 5–10)

The ANOVA revealed that the pace score for homozygotes for the *A* allele (*AA*) at the top SNP on ECA20 (N = 293, LSmeans = 7.15, se = ± 0.10) differed significantly (*p*-value < 0.001) from that of horses with the *AC* or *CC* genotype (N = 69, LSmeans = 6.26, se = ± 0.19). Mean scores for the other gaits assessed at breeding field tests were not significantly different between these two groups.

**Fig. 5:**
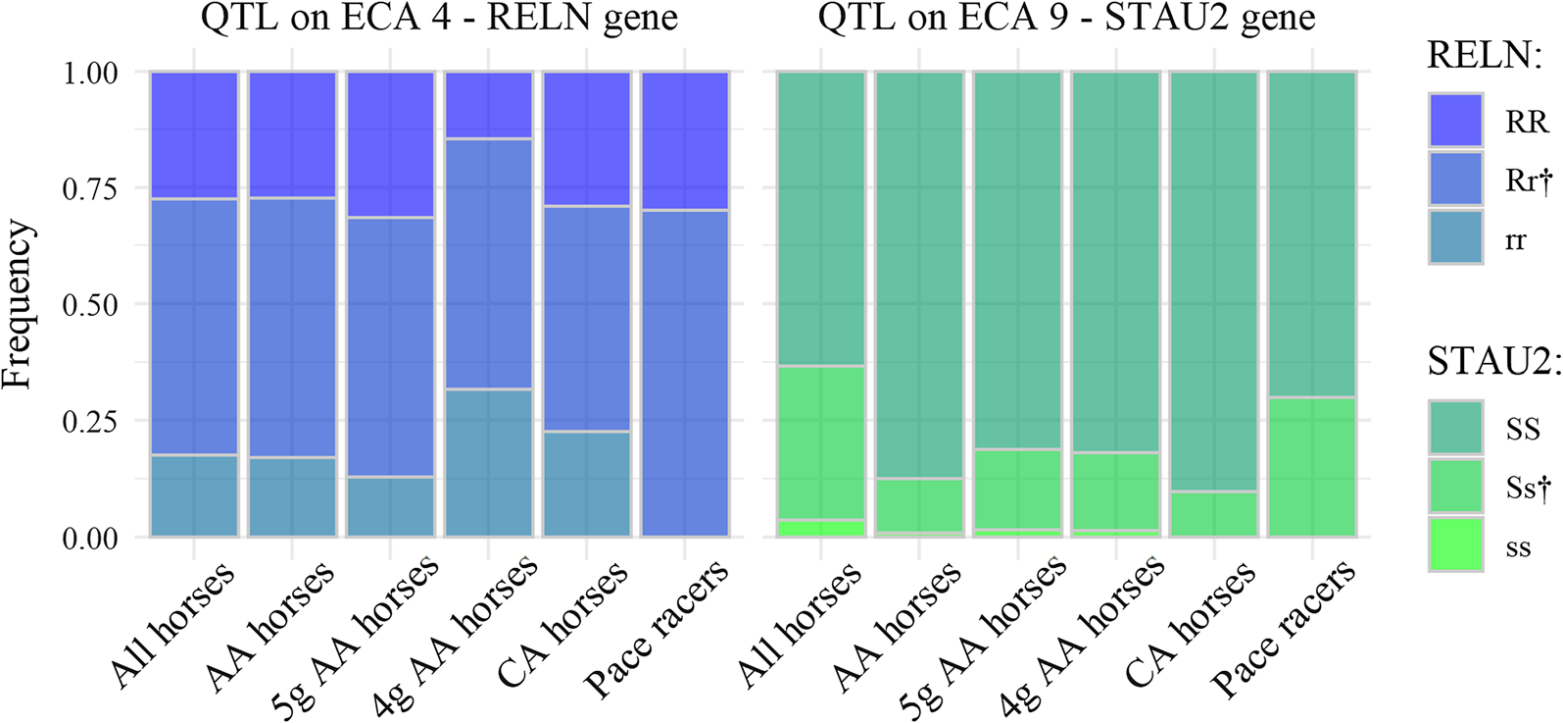
Frequencies of the genotypes at the *RELN* and *STAU2* genes in groups of horses with variable pacing ability. The number of horses within each group is All horses N = 372, *AA* horses N = 340, 5-gaited *AA* horses N = 248, 4-gaited *AA* horses N = 82, *CA* horses N = 31, and Pace racers N = 10. ^†^The frequency of the heterozygous genotypes (Rr and Ss) also included the rare haplotypes according to the haplotype analyses (1% of the sample size on ECA4 and 2% on ECA9)

We found that the genotype frequencies for the QTL detected in the *RELN* and *STAU2* genes differed between the horses that had different levels of pacing ability (Fig. 5). The group of horses with a genetic precondition to pace that had shown pace at breeding field tests (5-gaited *DMRT3 AA* horses) had higher frequencies of the RR and SS genotype at the *RELN* and *STAU2* genes, respectively, and a lower frequency of the rr genotype compared to all horses with the *AA* genotype at the *DMRT3* gene. On the contrary, the group of horses with a genetic precondition to pace that had not shown pace at breeding field tests (4-gaited *DMRT3 AA* horses) had the highest frequency of both the rr and ss genotypes of all groups of horses and the lowest frequency of the RR and SS genotypes.

The group of horses without the favorable genetic variant for pace (*DMRT3 CA* horses) had the highest frequency of the SS genotype among all groups, but a lower frequency of the heterozygous genotypes (Rr and Ss) and lacked the ss genotype altogether. The group of horses that had raced in pace competitions but that had not attended a breeding field test (pace racers) had a higher frequency of the heterozygous genotypes (Rr and Ss) compared to the other groups and lacked the rr and ss genotypes altogether.

For both QTL, genotype frequencies in the group of four-gaited *DMRT3 AA* horses differed significantly from those in the group of five-gaited *DMRT3 AA* horses (*p* < 0.001), the group of all *DMRT3 AA* horses (*p* = 0.015), and the group with all horses in the dataset (*p* = 0.015 (*RELN*), *p* = 0.008 (*STAU2*)). Pairwise comparison of genotype frequencies between other groups were non-significant (*p* > 0.05). The number of horses with different genotypes in each group is presented in Additional file 9: Table S4.

**Fig. 6:**
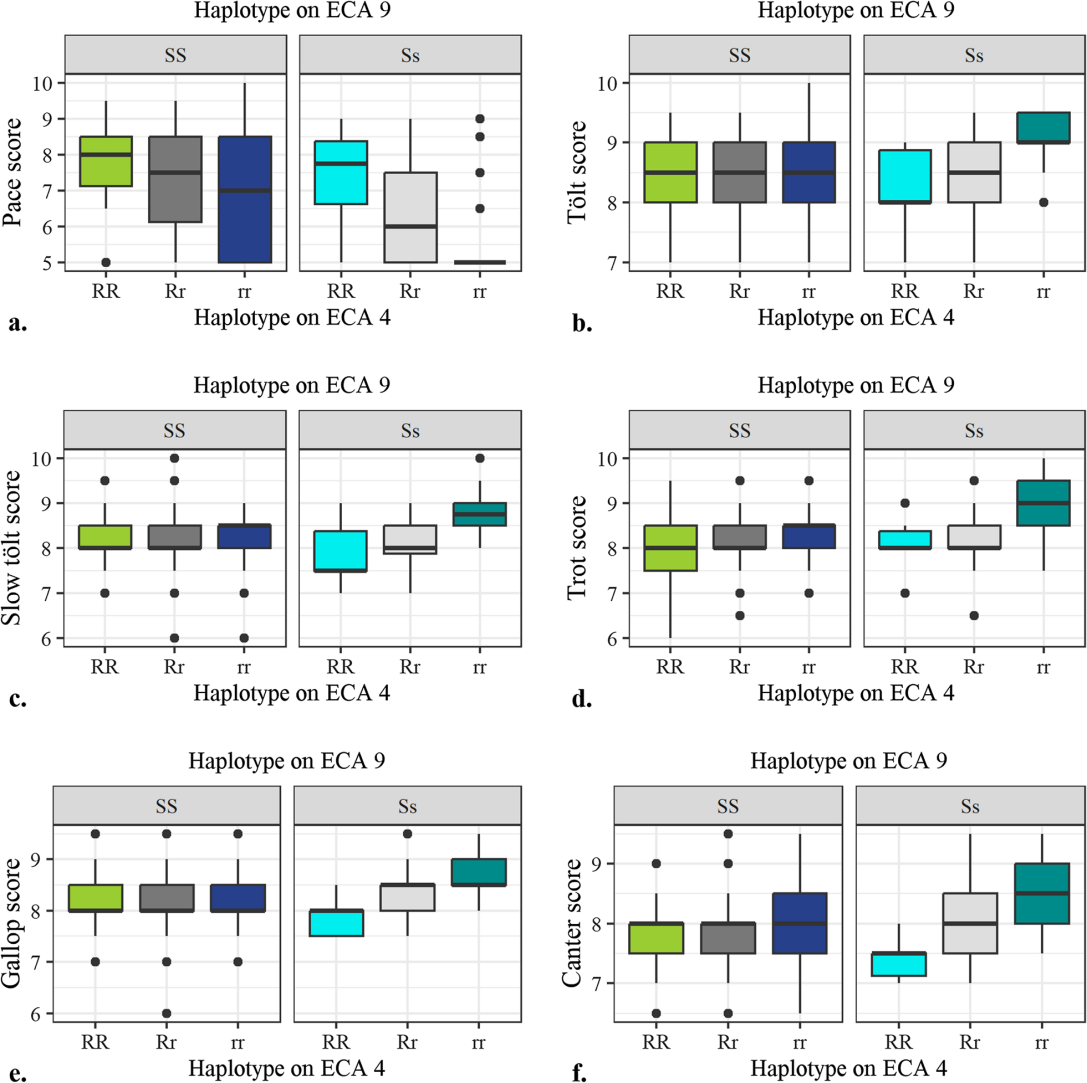
Interactions between the significant haplotypes on ECA4 and ECA9. Interactions between the significant haplotypes on ECA4 and ECA9 in a group of horses with the *AA* genotype at the *DMRT3*-variant on the subjective scores for **a** pace, **b** tölt, **c** slow tölt, **d** trot, **e** gallop and **f** canter. Number of horses with each combination is SS:RR = 78, SS:Rr = 150, SS:rr = 41, Ss:RR = 10, Ss:Rr = 29, Ss:rr = 17. The groups referred to as heterozygous individuals (Rr and Ss) also included horses possessing the rare haplotypes detected in the haplotype analyses (1% of the sample size with the rare haplotypes on ECA4 and 2% of the sample size with the rare haplotypes on ECA9)

Interactions between the significant haplotypes in the *RELN* gene on ECA4 and the *STAU2* gene on ECA9 were investigated within the group of horses with the *AA* genotype at the *DMRT3*-variant (Fig. 6). Horses with the SS and RR genotypes (SS:RR) had significantly higher mean scores for pace than horses with the Ss:Rr and Ss:rr genotype combinations. Horses with the Ss:rr genotype combination also had significantly lower mean scores for pace than those with the SS:Rr and SS:rr genotype combinations.

For the other gaits investigated (except walk), horses with the Ss:rr genotype combination had higher mean assessment scores than horses with the SS:RR and SS:Rr genotype combinations. Furthermore, horses with the Ss:rr genotype combination had higher mean scores than horses with the Ss:RR genotype combination for tölt, slow tölt, gallop, and canter. Horses with the Ss:rr genotype combination also had higher mean scores than horses with the Ss:Rr genotype combination for tölt and slow tölt, and than horses with the SS:rr genotype combination for slow tölt.

**Fig. 7:**
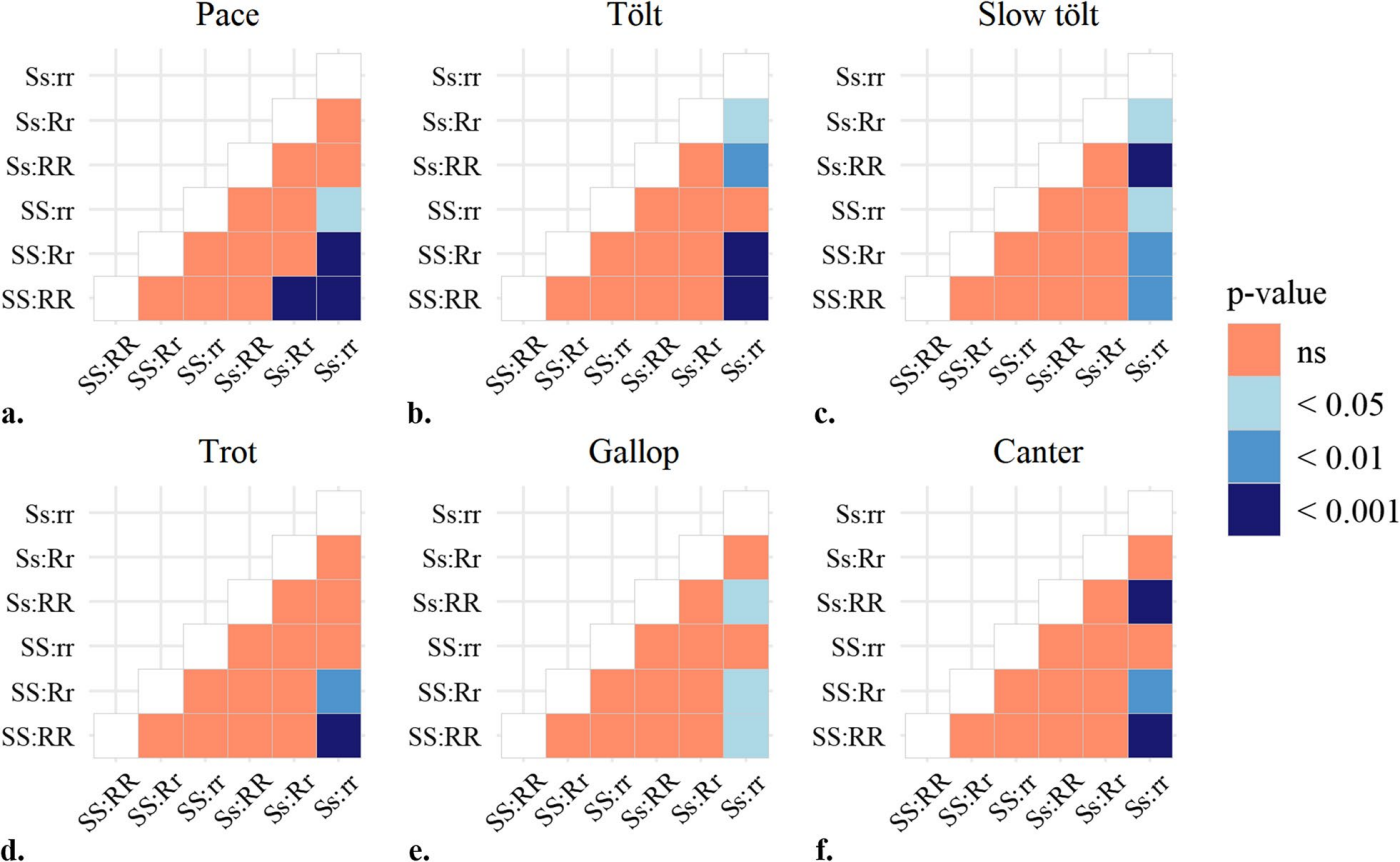
Significance levels of the differences in mean scores between different haplotype interactions for different gaits: **a** pace, **b** tölt, **c** slow tölt, **d** trot, **e** gallop, and **f** canter. Number of horses with each combination is SS:RR = 78, SS:Rr = 150, SS:rr = 41, Ss:RR = 10, Ss:Rr = 29, Ss:rr = 17

Results for horses with the ss:RR and ss:Rr genotype combinations (only 2 and 3 individuals, respectively) are not presented in Fig. 6, since the mean scores for these few horses did not differ significantly from the mean scores for horses with different genotype combinations. The significance levels of the differences in mean scores for other genotype interactions are presented in Fig. 7.

Analysis of the significant haplotypes in the *RELN* gene on ECA4 and the *STAU2* gene on ECA9 was also performed for all gaits within the group of 31 horses with the *CA* genotype at the *DMRT3*-variant, but these results were not significant (results not shown). In addition, we did not find clear indications of interactions between the haplotypes in the *RELN* and *STAU2* genes and the QTL previously detected on ECA22 for back and croup (results not shown).

## Discussion

The present study was performed as a first step towards a better understanding of the genetic architecture of pace beyond the contribution of the *DMRT3* gene by identifying other genomic regions that are relevant for this gait. A single associated SNP on ECA20 was located within a long non-coding RNA gene, in the vicinity of several other genes and its potential influence on pace is still unclear. Two novel QTL were detected on ECA4 and 9, which were located within the *RELN* and *STAU2* genes, respectively. Our results suggest that these QTL may be important not only for pace but also for other gaits that are assessed at breeding field tests and, to some extent, they seem to differentiate horses with different levels of pacing ability and quality. Furthermore, there seem to be some interactions between these QTL and the *DMRT3* gene, and genetic compensation may potentially play a role for this trait.

### Potential causative genes

While the two identified QTL on ECA4 and 9 are located in different genes, to date, we cannot rule out an effect on regulatory elements that govern the expression of nearby genes. However, as discussed below, both the *RELN* and *STAU2* genes appear to be likely candidates, whereas the genes in the vicinity of the identified regions (see Additional file 7: Table S3) are either all involved in general cellular processes or not expressed in neural tissue, making it unlikely that they affect development or the function of the locomotor network.

The QTL detected on ECA4 is located within the *RELN* gene, which encodes an extracellular matrix protein that mediates cell to cell interactions that are critical for migration and positioning of neurons during development [39–42]. The *RELN* gene is expressed in the developing spinal cord of zebrafish and mice but its expression has also been described in the mature spinal cord of primates, which indicates a vital and conserved function during spinal cord development [43]. The *RELN* gene has been widely explored and several mutations have been reported in mice and rats that are associated with the “reeler phenotype” [44–48], which results in abnormal locomotor behaviors such as tremors, dystonia, and ataxia [48]. Abnormal migration of motor neurons has been observed in mutant mice, which likely affects their synaptic input and might be responsible for the reeler phenotype [41, 49]. Given that neurons expressing the *DMRT3* gene are known to synapse onto motor neurons [7, 50], the normal inhibition provided by these neurons could be disturbed due to aberrant innervation, thus explaining the cumulative effect of the ‘gait keeper’ mutation and the RR genotype.

The QTL on ECA9 is located within the *STAU2* gene, which encodes a protein involved in mRNA transport [51]. The *STAU2* gene is expressed in the developing zebrafish and in the mouse spinal cord, and contributes to the asymmetric distribution of mRNA in dividing intermediate progenitor cells [52], which is a process known to affect the proliferation and fate assignment of neurons. Loss of *STAU2* affects the survival of neurons and mutant mice display reduced motor coordination but enhanced motor learning abilities [53]. This suggests that, while negatively affecting early motor output, it may facilitate the learning of new gaits, as in the case of five-gaited horses. Thus, modification of the expression of *STAU2* may improve the ability of Icelandic horses to learn to pace, which may explain the higher quality pace in horses that are homozygous for the ‘gait keeper’ mutation and that also carry the SS genotype.

### Effects of haplotypes in the *RELN* gene on gaits and topline conformation

Breeding horses are preselected to attend breeding field tests based on their assumed potential to get high scores, where the emphasis on tölt and pace is strong, in parallel to their importance in the breeding goal [10, 11]. Therefore, the somewhat balanced frequency of the opposite haplotypes at the *RELN* gene (0.54 for R and 0.45 for r) is not unexpected, considering their opposite effects on pace and tölt. Furthermore, horses that are preselected to be shown at breeding field tests despite a poor or absent ability to pace tend to have higher average quality for the other gaits to compensate for the missing score for pace. This could partly explain the higher average scores for all gaits except pace of horses with the rr genotype, since the four-gaited horses with the *CA* or *AA* genotype at the *DMRT3*-variant were more likely to have the rr genotype than the other horses in the study. However, favorable effects of the r haplotype on tölt, trot, gallop, and canter cannot be completely excluded. The relatively high frequency of the R haplotype in horses with the *AA* genotype at the *DMRT3*-variant that do perform pace, either at breeding field tests or in pace competitions, further supports the cumulative effect of the R haplotype and the ‘gait keeper’ mutation for pacing ability and quality.

The QTL in the *RELN* gene seems to affect not only the gait traits but also morphological traits. Horses with the rr genotype had on average higher withers and were higher at the front compared to horses with the RR genotype. The advantage of high withers and an uphill conformation for riding ability in Icelandic horses has been previously reported [14]. However, the relationship between score for pace and height of withers, as well as height at the front, was curvilinear, which indicates that there may be an optimum height of withers and height at the front for the pace score. This may contribute to the opposite effects of the identified QTL in the *RELN* gene on pace score and scores for the other gaits assessed at breeding field tests. On the other hand, the preselection of individuals for a higher quality of tölt, trot, gallop, and canter among the horses shown without pace at breeding field tests, may also promote bias in the data in that horses performing as four-gaiters have higher withers and a more uphill conformation as they have been shown to be favorable attributes for those gaits.

### Haplotypes in the *STAU2* gene may influence locomotive learning

The high frequency of the S haplotype at the *STAU2* gene indicates a strong selection for this genomic region. This is supported by the large favorable effect of the S haplotype on pace, which has been strongly emphasized in the breeding goal since 1950 [54]. The other lateral gait, tölt, has also been emphasized in the breeding goal and has been reported to have a favorable, but not especially high, genetic correlation (0.38) with pace [12]. However, the results from our present study suggest rather an unfavorable effect of this haplotype on all gaits except pace, particularly on trot and gallop. These effects of the S haplotype concur to some extent with the observed effects of the *A* allele at the *DMRT3* gene [7, 8]. This may indicate a similar reinforcement by the S haplotype to that of the *A* allele in *DMRT3* for the coordination of the ipsilateral legs and subsequent negative effect on the synchronized movement of diagonal legs, or possibly cumulative effects of the two genetic factors.

The high frequency of the SS genotype (90%) in the group of horses with the *CA* genotype at the *DMRT3*-variant suggests that the S haplotype may be important for *CA* horses to perform well at breeding field tests. The S haplotype may improve motor learning abilities, similar to the effects of a variant in the *STAU2* gene in mice [53]. In our dataset, based only on their *DMRT3* genotype, few of the horses that carried the SS genotype would have been expected not to receive the scores for tölt or pace that they got at breeding field tests. One horse with the *CC* genotype at the *DMRT3*-variant received a score of 7.5 for tölt and two horses with the *CA* genotype received scores of 6.0 and 6.5 for pace (results not shown). The number of such horses is too small to draw firm conclusions, but further studies on whether the S haplotype could compensate for the lack of the *A*-allele (*DMRT3*) would be interesting.

### Genetic difference between five-gaited horses and pace racers

Both the R haplotype at the *RELN* gene and the S haplotype at the *STAU2* gene showed large favorable effects on pace performance at breeding field tests. Therefore, it is interesting to compare the group of five-gaited horses with the more specialized pace racers. Good pace racers need to have the ability to pace in balance at high speed, but in contrast to the five-gaited horses shown at breeding field tests, the quality of the other gaits is less important for racing performance. Therefore, not all pace racers are good five-gaited horses and vice versa, which may be traced back to different genetic makeups of the two groups, in addition to different training methods. The most prominent genetic difference between these two groups that was observed in the current study was the absence of the rr genotype among the pace racers and the relatively high frequency of this genotype among the five-gaiters. This may further indicate the importance of the R haplotype for pacing ability, but the r haplotype may also enhance the quality of the other gaits, which is more important for the five-gaited horse. However, there were only ten pace racers included in this study, making the comparison somewhat uncertain. Thus, further research with a larger number of pace racers is needed to validate the genetic difference between pace racers and five-gaited horses used for other purposes.

### Interactions between haplotypes in the *RELN* and *STAU2* genes

The RR:SS genotype combination was the most favorable for pace score, which indicates that this genotype combination has additive effects. In contrast, the rr:Ss genotype combination was the most favorable for scores for tölt, slow tölt, trot, gallop, and canter. This is consistent with the individual effects of the haplotypes, assuming that the r haplotype enhances the quality of the gaits other than pace and the S haplotype enhances motor learning.

### Data quality

In general, genetic studies rely heavily on the quality of the phenotypes used in the analysis. Objective measurements are usually less affected by environmental factors and therefore tend to yield higher heritability estimates. However, for routine large-scale recordings in field tests of horses, gait traits are, for practical and economic reasons, still more often subjectively than objectively assessed. The moderate to high heritability estimates for gait scores in Icelandic horses obtained in several studies [10, 12], in particular for pace, as well as results from previous genomic studies [7, 15] show that the standardized assessments by trained judges do provide useful information. In this study, we used the effects accounted for in the current statistical model for the routine genetic evaluation of Icelandic horses as a starting point, as well as some additional environmental factors such as the *DMRT3* genotype and the country where the horse was assessed. In our dataset of genotyped horses, only the effects of sex and *DMRT3* genotype were significant.

The quality of our data is furthermore subject to the relationships between the individuals in our sample, since the Icelandic horse population is a relatively small and closed population [55]. However, according to the MDS plot (Fig. 3), which was based on the genomic-kinship matrix from our dataset, no stratification or outliers were detected. Furthermore, relationships between individuals were accounted for by including the genomic-kinship matrix in the GWA model. Therefore, we do not believe that the results were affected by bias due to relationships between individuals.

## Conclusions

This study provides valuable information about the genetic background of the gait pace in Icelandic horses. Two new candidate genes, *RELN* and *STAU2*, were identified through GWAS. Both genes are known to be associated with locomotor behavior in mice, which may indicate analogous functions in horses. Furthermore, the results from this study indicate genetic interactions between these novel genes and the previously identified candidate gene *DMRT3*. The novel genes and the identified top SNP on ECA20, along with the effects of *DMRT3* genotype were estimated to account for 27.4% of the phenotypic variance of the gait. Opposite haplotypes were identified for both these genes that appear to influence quality and ability to pace. Furthermore, these haplotypes appear to have opposing effects on the other gaits, especially trot, gallop, and canter. This suggests that these genetic factors may partly contribute to whether a horse is trained and shown as a four- or five-gaited horse at a breeding field test. These findings may aid in the selection of breeding and competition horses and are thus of major interest to horse breeders. Further investigation is needed to fully understand the underlying genetic factors and the nature of their interactions that contribute to the complexity of the pace trait’s phenotype.

### Supplementary Information

**Additional file 1: Figure S1.** Distributions of scores for pace after rank- and log-transformation. (a) Rank- (W = 0.98) and (b) log-transformed (W = 0.90) distribution of scores for pace in the sample of 362 horses assessed at a breeding field test. Neither of these transformations resulted in a normal distribution of the scores (*p* > 0.05)

**Additional file 2: Table S1.** Descriptive statistics of the gait scores (other than pace). Number of assessments, mean score, standard deviation (sd), range, skewness, kurtosis, and a *p*-value from a Jarque–Bera Normality Test for the gaits (other than pace) assessed at breeding field tests for horses included in the dataset.

**Additional file 3: Figure S2.** Distribution of the gait scores (other than pace). Distribution of scores for (a) tölt, (b) slow tölt, (c) trot, (d) gallop, (e) canter, and (f) walk in the sample of 362 horses assessed at a breeding field test.

**Additional file 4: Figure S3.** Distributions of the scores for tölt after rank- and log-transformation. (a) Rank- (W = 0.98) and (b) log-transformed (W = 0.95) distribution of scores for tölt in the sample of 362 horses assessed at a breeding field test. Neither of these transformations resulted in a normal distribution of the scores (*p* > 0.05).

**Additional file 5: Table S2.** Top 50 SNPs from the GWAS. A summary of the GWAS results for the 50 top SNPs.

**Additional file 6: Figure S4.** Relative location of the identified SNPs in the *RELN* horse gene.

**Additional file 7: Table S3.** List of genes located in the vicinity of the regions identified on ECA4, 9, and 20. A list of all the genes located in the vicinity (± 500.00 kb) of the regions identified on ECA4: 4,222,615–4,228,914 bp [39–42, 56–64], ECA9: 11,533,922–13,457,268 bp [31, 32, 51, 52, 65–72] and ECA20: 52,057,378–52,057,378 bp.

**Additional file 8: Figure S5.** Relative location of the identified SNPs in the *STAU2* horse gene. Relative location of the identified SNPs in the *STAU2* horse gene. Two of the identified SNPs, that were located ~ 1600 kb away from the gene, were not included in this figure.

**Additional file 9: Table S4.** Number of horses within each group of horses with variable pacing ability. The number of horses with different haplotypes on ECA4 and ECA9 in groups of horses with variable pacing ability.

## Data Availability

The datasets generated and analyzed during the current study are not publicly available since the study was performed in collaboration with the Icelandic horse breeding industry and has commercial value for them. However, data are available from the corresponding author on reasonable request and with the permission of the Icelandic Horse Association.
