# Supplementary figures and images for: The genetics of gaits in Icelandic horses goes beyond DMRT3, with RELN and STAU2 identified as two new candidate genes
Source: Genet Sel Evol. 2023 Dec 11;55:89. doi: 10.1186/s12711-023-00863-6 (PMC10712087; doi:10.1186/s12711-023-00863-6)

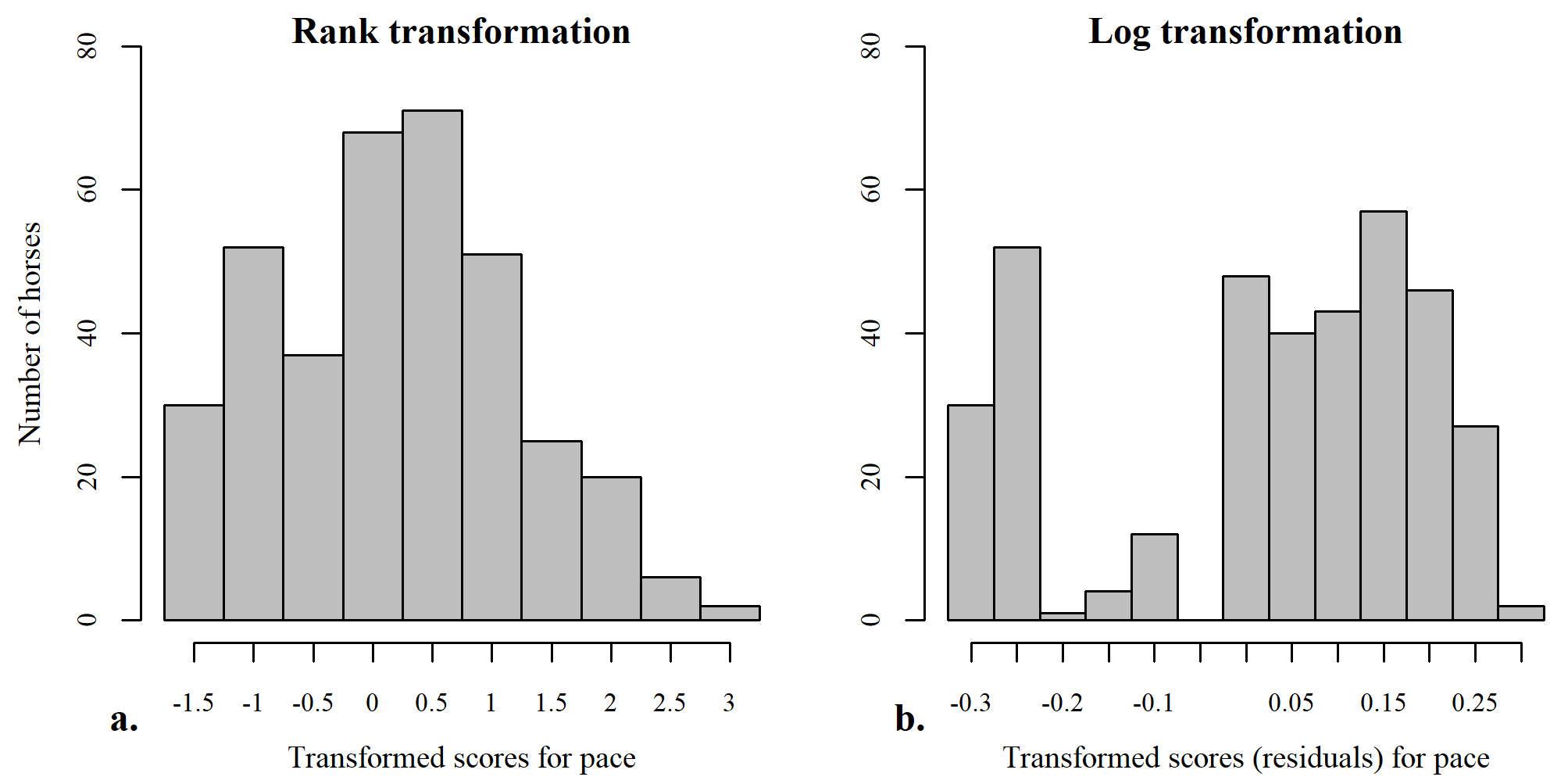

Supplement: Supplementary file 1 — Additional file 1: Figure S1. Distributions of scores for pace after rank- and log-transformation. (a) Rank- (W = 0.98) and (b) log-transformed (W = 0.90) distribution of scores for pace in the sample of 362 horses assessed at a breeding field test. Neither of these transformations resulted in a normal distribution of the scores (p > 0.05) [file 12711_2023_863_MOESM1_ESM.png]

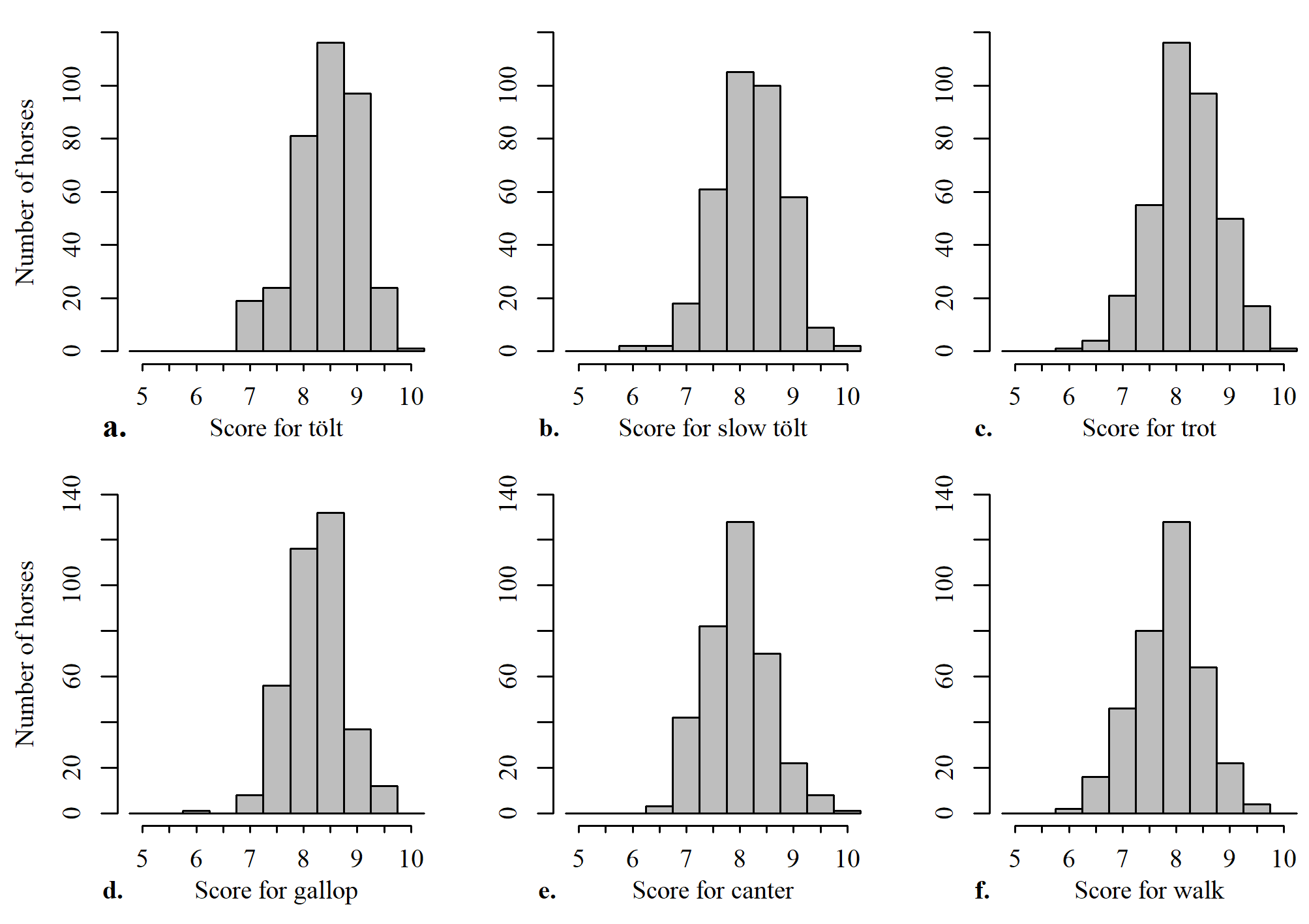

Supplement: Supplementary file 3 — Additional file 3: Figure S2. Distribution of the gait scores (other than pace). Distribution of scores for (a) tölt, (b) slow tölt, (c) trot, (d) gallop, (e) canter, and (f) walk in the sample of 362 horses assessed at a breeding field test. [file 12711_2023_863_MOESM3_ESM.png]

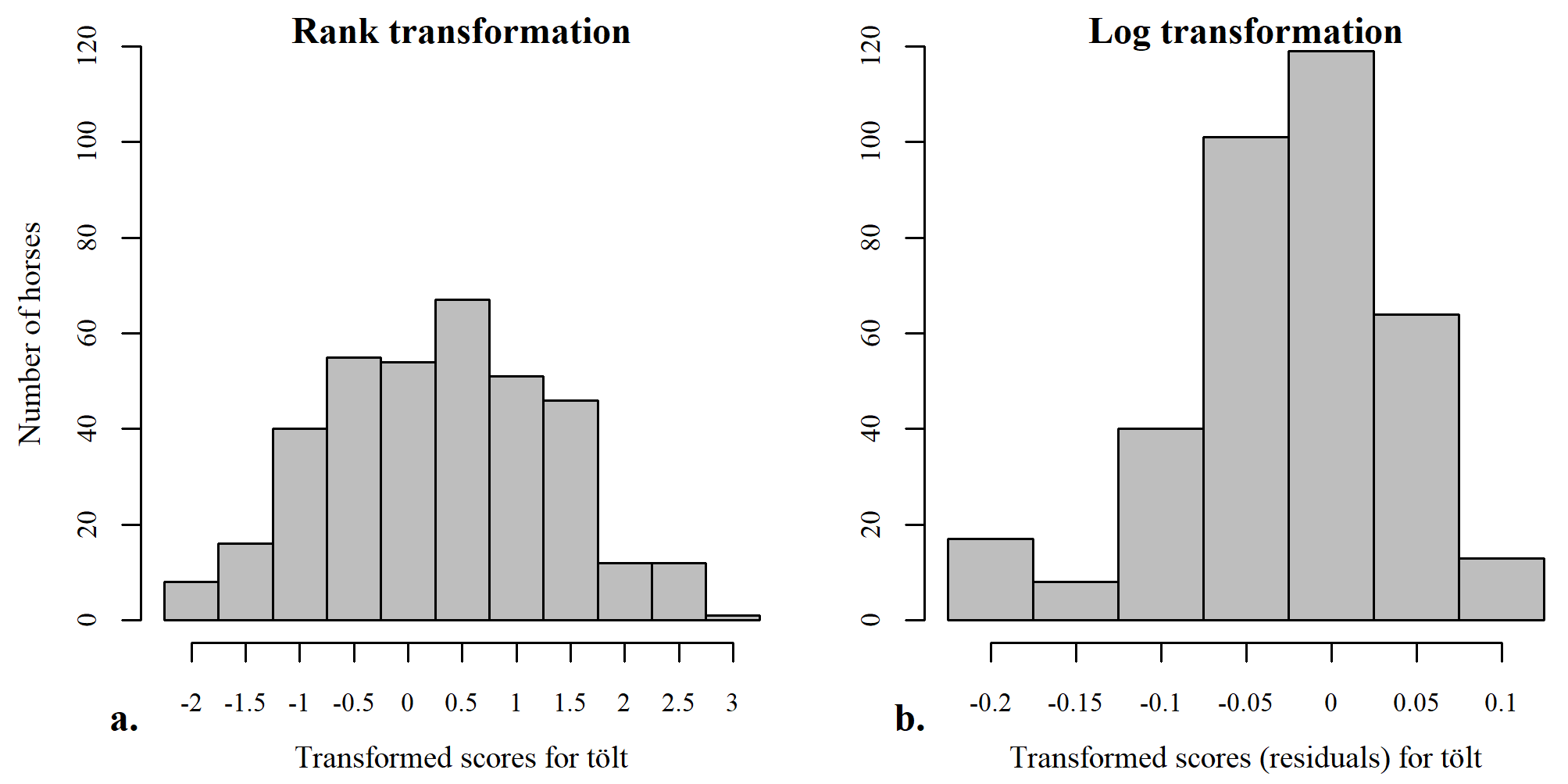

Supplement: Supplementary file 4 — Additional file 4: Figure S3. Distributions of the scores for tölt after rank- and log-transformation. (a) Rank- (W = 0.98) and (b) log-transformed (W = 0.95) distribution of scores for tölt in the sample of 362 horses assessed at a breeding field test. Neither of these transformations resulted in a normal distribution of the scores (p > 0.05). [file 12711_2023_863_MOESM4_ESM.png]

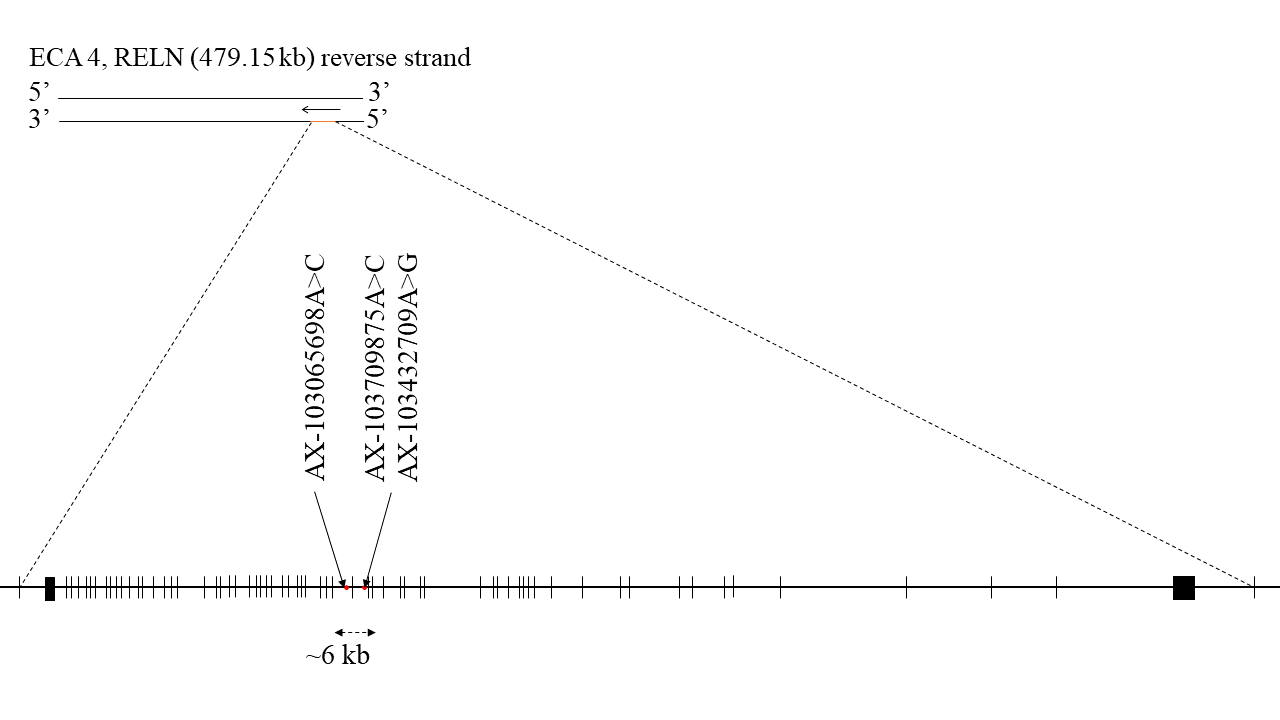

Supplement: Supplementary file 6 — Additional file 6: Figure S4. Relative location of the identified SNPs in the RELN horse gene. [file 12711_2023_863_MOESM6_ESM.png]

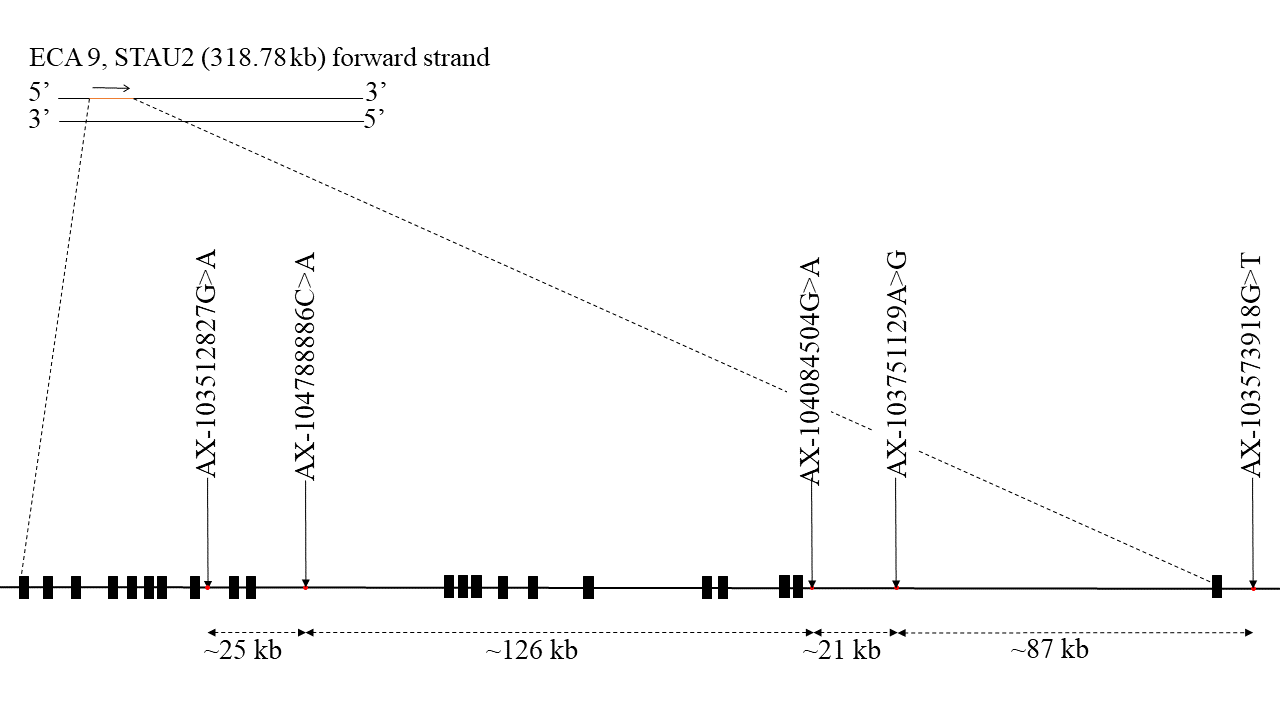

Supplement: Supplementary file 8 — Additional file 8: Figure S5. Relative location of the identified SNPs in the STAU2 horse gene. Relative location of the identified SNPs in the STAU2 horse gene. Two of the identified SNPs, that were located ~ 1600 kb away from the gene, were not included in this figure. [file 12711_2023_863_MOESM8_ESM.png]
