# Supplementary material for: The genetics of gaits in Icelandic horses goes beyond DMRT3, with RELN and STAU2 identified as two new candidate genes
Source: Genet Sel Evol. 2023 Dec 11;55:89. doi: 10.1186/s12711-023-00863-6 (PMC10712087; doi:10.1186/s12711-023-00863-6)
Supplement: Supplementary file 2 — Additional file 2: Table S1. Descriptive statistics of the gait scores (other than pace). Number of assessments, mean score, standard deviation (sd), range, skewness, kurtosis, and a p-value from a Jarque–Bera Normality Test for the gaits (other than pace) assessed at breeding field tests for horses included in the dataset. [file 12711_2023_863_MOESM2_ESM.docx]

| **Gait** | **No. of assessments** | **Mean** | **sd** | **Range** | **Skewness** | **Kurtosis** | **Jarque-Bera Normality Test (*p*-value)** |
| --- | --- | --- | --- | --- | --- | --- | --- |
| Tölt | 362 | 8.45 | ±0.61 | 7.0-10.0 | -0.44 | 2.92 | 0.003 |
| Slow tölt | 357 | 8.20 | ±0.63 | 6.0-10.0 | -0.20 | 3.25 | 0.186 |
| Trot | 362 | 8.19 | ±0.65 | 6.0-10.0 | -0.12 | 3.08 | 0.634 |
| Gallop | 362 | 8.23 | ±0.54 | 6.0-9.5 | -0.09 | 3.49 | 0.131 |
| Canter | 356 | 7.95 | ±0.61 | 6.5-10.0 | 0.27 | 3.11 | 0.100 |
| Walk | 362 | 7.85 | ±0.64 | 6.0-9.5 | -0.15 | 2.96 | 0.487 |
