# Supplementary material for: The genetics of gaits in Icelandic horses goes beyond DMRT3, with RELN and STAU2 identified as two new candidate genes
Source: Genet Sel Evol. 2023 Dec 11;55:89. doi: 10.1186/s12711-023-00863-6 (PMC10712087; doi:10.1186/s12711-023-00863-6)
Supplement: Supplementary file 5 — Additional file 5: Table S2. Top 50 SNPs from the GWAS. A summary of the GWAS results for the 50 top SNPs. [file 12711_2023_863_MOESM5_ESM.docx]

| **SNP id** | **Chr. no.** | **Position** | **Strand** | **A1** | **A2** | **N** | **effB** | **se_effB** | **chi2.1df** | **P1df** | **Pc1df** |
| --- | --- | --- | --- | --- | --- | --- | --- | --- | --- | --- | --- |
| AX-104084504 | 9 | 13349141 | u | A | G | 362 | -0,97 | 0,18 | 27,52 | 1,55E-07 | 1,55E-07 |
| AX-103512827 | 9 | 13198591 | u | A | G | 362 | -0,95 | 0,19 | 24,58 | 7,12E-07 | 7,12E-07 |
| AX-103751129 | 9 | 13370069 | u | G | A | 359 | -0,92 | 0,19 | 24,40 | 7,81E-07 | 7,81E-07 |
| AX-104788886 | 9 | 13223377 | u | A | C | 362 | -0,91 | 0,19 | 23,10 | 1,54E-06 | 1,54E-06 |
| AX-103999707 | 20 | 52057378 | u | A | C | 362 | -0,86 | 0,18 | 22,72 | 1,87E-06 | 1,87E-06 |
| AX-103709875 | 4 | 4228503 | u | C | A | 360 | -0,52 | 0,11 | 21,13 | 4,30E-06 | 4,30E-06 |
| AX-103573918 | 9 | 13457268 | u | T | G | 362 | -0,93 | 0,20 | 20,81 | 5,07E-06 | 5,07E-06 |
| AX-103065698 | 4 | 4222615 | u | C | A | 361 | -0,51 | 0,11 | 20,56 | 5,77E-06 | 5,77E-06 |
| AX-103968085 | 9 | 11533922 | u | C | T | 361 | -0,77 | 0,17 | 20,32 | 6,56E-06 | 6,56E-06 |
| AX-103082371 | 9 | 11539183 | u | G | A | 361 | -0,76 | 0,17 | 19,80 | 8,61E-06 | 8,61E-06 |
| AX-103432709 | 4 | 4228914 | u | G | A | 358 | 0,50 | 0,11 | 19,65 | 9,30E-06 | 9,30E-06 |
| AX-103042739 | 9 | 11900528 | u | A | G | 361 | -0,80 | 0,18 | 19,51 | 1,00E-05 | 1,00E-05 |
| AX-104450574 | 9 | 11421551 | u | A | G | 359 | -0,74 | 0,17 | 18,60 | 1,62E-05 | 1,62E-05 |
| AX-103083747 | 17 | 5552199 | u | G | A | 361 | -0,47 | 0,11 | 18,50 | 1,70E-05 | 1,70E-05 |
| AX-103167744 | 23 | 1086472 | u | T | C | 362 | 0,44 | 0,10 | 18,37 | 1,82E-05 | 1,82E-05 |
| AX-103165651 | 9 | 11466113 | u | C | T | 361 | -0,74 | 0,17 | 18,25 | 1,94E-05 | 1,94E-05 |
| AX-105002058 | 12 | 8320946 | u | C | A | 362 | 0,51 | 0,12 | 17,98 | 2,24E-05 | 2,24E-05 |
| AX-103317392 | 4 | 5927014 | u | G | T | 362 | -0,80 | 0,19 | 17,71 | 2,57E-05 | 2,57E-05 |
| AX-104422036 | 23 | 1051786 | u | A | G | 354 | 0,44 | 0,11 | 17,51 | 2,87E-05 | 2,87E-05 |
| AX-104797678 | 5 | 22954712 | u | C | T | 360 | 0,49 | 0,12 | 17,45 | 2,95E-05 | 2,95E-05 |
| AX-103154296 | 23 | 1052808 | u | C | T | 361 | 0,43 | 0,10 | 17,30 | 3,19E-05 | 3,19E-05 |
| AX-103107550 | 19 | 27377209 | u | A | G | 362 | -0,53 | 0,13 | 17,21 | 3,34E-05 | 3,34E-05 |
| AX-104097144 | 8 | 65126384 | u | T | C | 361 | 0,46 | 0,11 | 17,13 | 3,50E-05 | 3,50E-05 |
| AX-103801397 | 17 | 5424487 | u | G | A | 356 | -0,46 | 0,11 | 17,12 | 3,51E-05 | 3,51E-05 |
| AX-104248939 | 12 | 8887787 | u | G | A | 362 | -0,50 | 0,12 | 16,95 | 3,83E-05 | 3,83E-05 |
| AX-104937620 | 20 | 52063814 | u | G | T | 362 | -1,05 | 0,25 | 16,95 | 3,84E-05 | 3,84E-05 |
| AX-104579833 | 20 | 52119690 | u | C | T | 362 | -1,05 | 0,25 | 16,95 | 3,84E-05 | 3,84E-05 |
| AX-104958809 | 20 | 52157614 | u | G | A | 362 | -1,05 | 0,25 | 16,95 | 3,84E-05 | 3,84E-05 |
| AX-104239105 | 20 | 52166487 | u | G | A | 361 | -1,04 | 0,25 | 16,81 | 4,14E-05 | 4,14E-05 |
| AX-103510578 | 16 | 39091302 | u | C | T | 361 | -0,51 | 0,12 | 16,77 | 4,23E-05 | 4,23E-05 |
| AX-104197284 | 1 | 88182475 | u | C | T | 362 | 0,47 | 0,12 | 16,69 | 4,41E-05 | 4,41E-05 |
| AX-104304328 | 16 | 39218559 | u | T | C | 362 | -0,51 | 0,12 | 16,67 | 4,44E-05 | 4,44E-05 |
| AX-103735133 | 3 | 59324339 | u | G | T | 362 | -0,62 | 0,15 | 16,62 | 4,58E-05 | 4,58E-05 |
| AX-104732396 | 17 | 3936253 | u | T | C | 353 | -0,47 | 0,12 | 16,52 | 4,82E-05 | 4,82E-05 |
| AX-104253394 | 9 | 11450827 | u | G | A | 357 | -0,71 | 0,17 | 16,45 | 4,98E-05 | 4,98E-05 |
| AX-104088419 | 17 | 5157739 | u | A | G | 360 | 0,48 | 0,12 | 16,12 | 5,95E-05 | 5,95E-05 |
| AX-103521488 | 30 | 19974830 | u | T | C | 362 | 0,81 | 0,20 | 16,11 | 5,97E-05 | 5,97E-05 |
| AX-104558538 | 30 | 17294132 | u | A | G | 362 | -0,54 | 0,13 | 16,04 | 6,19E-05 | 6,19E-05 |
| AX-104084790 | 16 | 41894723 | u | G | A | 362 | -0,47 | 0,12 | 15,72 | 7,35E-05 | 7,35E-05 |
| AX-102998130 | 16 | 38945590 | u | A | G | 362 | -0,49 | 0,12 | 15,62 | 7,75E-05 | 7,75E-05 |
| AX-103013990 | 17 | 7322132 | u | C | T | 355 | 0,46 | 0,12 | 15,51 | 8,21E-05 | 8,21E-05 |
| AX-103943022 | 4 | 5868085 | u | C | T | 362 | -0,44 | 0,11 | 15,40 | 8,71E-05 | 8,71E-05 |
| AX-103886952 | 5 | 37182242 | u | G | A | 362 | 0,45 | 0,12 | 15,40 | 8,72E-05 | 8,72E-05 |
| AX-104554994 | 17 | 70389665 | u | T | C | 362 | -0,43 | 0,11 | 15,37 | 8,85E-05 | 8,85E-05 |
| AX-102975681 | 23 | 1077668 | u | C | T | 361 | 0,40 | 0,10 | 15,27 | 9,30E-05 | 9,30E-05 |
| AX-104924137 | 3 | 59420994 | u | A | G | 360 | -0,54 | 0,14 | 15,26 | 9,39E-05 | 9,39E-05 |
| AX-104008105 | 5 | 60703874 | u | C | A | 362 | -0,78 | 0,20 | 14,99 | 1,08E-04 | 1,08E-04 |
| AX-104108814 | 5 | 60732451 | u | C | T | 362 | -0,78 | 0,20 | 14,99 | 1,08E-04 | 1,08E-04 |
| AX-104110601 | 5 | 60747788 | u | A | G | 362 | -0,78 | 0,20 | 14,99 | 1,08E-04 | 1,08E-04 |
| AX-103851184 | 5 | 60749754 | u | T | C | 362 | -0,78 | 0,20 | 14,99 | 1,08E-04 | 1,08E-04 |

SNP id: SNP/Probe identification acc. to EquCab3.0 reference genome

Chr. no.: Chromosome number

A1: Alternate allele

A2: Reference allele

N: Number of tested individuals

effB: Effect of the B allele in allelic test

se_effB: Standard error of the effect of the B allele in allelic test

chi2.1df: Chi-squared statistic of 1-d.f.

P1df: *p*-values of 1-d.f. (additive or allelic) test for association between SNP and trait

Pc1df: *p*-values from the 1-d.f. test for association between SNP and trait; the statistic is corrected for possible inflation
