# Supplementary material for: The genetics of gaits in Icelandic horses goes beyond DMRT3, with RELN and STAU2 identified as two new candidate genes
Source: Genet Sel Evol. 2023 Dec 11;55:89. doi: 10.1186/s12711-023-00863-6 (PMC10712087; doi:10.1186/s12711-023-00863-6)
Supplement: Supplementary file 7 — Additional file 7: Table S3. List of genes located in the vicinity of the regions identified on ECA4, 9, and 20. A list of all the genes located in the vicinity (± 500.00 kb) of the regions identified on ECA4: 4,222,615–4,228,914 bp [39–42, 56–64], ECA9: 11,533,922–13,457,268 bp [31, 32, 51, 52, 65–72] and ECA20: 52,057,378–52,057,378 bp. [file 12711_2023_863_MOESM7_ESM.docx]

| Gene name | Gene type | Description | ECA | Location | Function |
| --- | --- | --- | --- | --- | --- |
| *FBXL13* | Protein coding | F-box and leucine rich repeat protein 13 | 4 | 3,667,015-3,840,570 | Protein-ubiquitin ligase |
| *LRRC17* | Protein coding | leucine rich repeat containing 17 | 4 | 3,762,165-3,774,356 | Osteoblast differentiation |
| *ARMC10* | Protein coding | armadillo repeat containing 10 | 4 | 3,876,367-3,903,278 | Interacts with p53 |
| *NAPEPLD* | Protein coding | N-acyl phosphatidylethanolamine phospholipase D | 4 | 3,906,564-3,929,476 | Enzyme that catalyzes the release of N-acylethanolamine |
| *PMPCB* | Protein coding | peptidase, mitochondrial processing subunit beta | 4 | 3,984,502-3,999,284 | Catalyzes peptide cleavage in the mitochondria |
| *DNAJC2* | Protein coding | DnaJ heat shock protein family (Hsp40) member C2 | 4 | 3,999,413-4,029,727 | Molecular chaperone |
| *PSMC2* | Protein coding | proteasome 26S subunit, ATPase 2 | 4 | 4,032,246-4,055,177 | Protein degradation |
| *SLC26A5* | Protein coding | solute carrier family 26 member 5 | 4 | 4,060,040-4,105,131 | Molecular motor in outer hair cells of the cochlea |
| *ENSECAG00000043489* | Protein coding | - | 4 | 4,112,992-4,115,259 |  |
| *RELN* | Protein coding | reelin | 4 | 4,128,582-4,586,870 | Extracellular matrix glycoprotein that aid neuronal migration |
| *ORC5* | Protein coding | origin recognition complex subunit 5 | 4 | 4,637,172-4,762,487 | Initiation of DNA replication |
| *LY96* | Protein coding | lymphocyte antigen 96 | 9 | 12,867,353-12,934,859 | Involved in binding lipopolysaccharide with Toll-Like Receptor |
| *TMEM70* | Protein coding | transmembrane protein 70 | 9 | 12,939,470-12,946,533 | Involved in the assembly of ATP synthase in mitrocondria |
| *ELOC* | Protein coding | elongin C | 9 | 12,960,316-12,968,377 | Activates elongation by RNA polymerase II |
| *U6* | SnRNA | U6 spliceosomal RNA | 9 | 12,967,939-12,968,046 | The non-coding RNA component of U6 snRNP in the spliceosome |
| *UBE2W* | Protein coding | ubiquitin conjugating enzyme E2 W | 9 | 13,009,984-13,072,414 | Ubiquitin-conjugating enzyme |
| *STAU2* | Protein coding | staufen double-stranded RNA binding protein 2 | 9 | 13,154,734-13,452,168 | Double-stranded RNA-binding protein enriched in the nervous system |
| *Metazoa_SRP* | Misc RNA | Metazoan signal recognition particle RNA | 9 | 13,199,365-13,199,645 | RNA Gene affiliated with the misc RNA class |
| *ENSECAG00000028748* | LncRNA | - | 9 | 13,492,301-13,494,621 |  |
| *RDH10* | Protein coding | retinol dehydrogenase 10 | 9 | 13,531,957-13,565,714 | Embryonic RA biosynthesis |
| *RPL7* | Protein coding | ribosomal protein L7 | 9 | 13,566,996-13,570,156 | 60S ribosomal protein |
| *ENSECAG00000031944* | Protein coding | - | 9 | 13,575,304-13,619,979 |  |
| *ENSECAG00000038059* | LncRNA | - | 9 | 13,712,729-13,715,351 |  |
| *SBSPON* | Protein coding | somatomedin B and thrombospondin type 1 domain containing | 9 | 13,731,642-13,753,743 | Predicted to be an extracellular matrix structural constituent |
| *TERF1* | Protein coding | telomeric repeat binding factor 1 | 9 | 13,764,488-13,800,361 | Telomere specific protein |
| *GSTA3* | Protein coding | glutathione S-transferase alpha 3 | 20 | 51,364,268-51,625,240 |  |
| *ENSECAG00000051447* | Protein coding | - | 20 | 51,587,188-51,628,359 |  |
| *ENSECAG00000053489* | Protein coding | - | 20 | 51,680,775-51,698,011 |  |
| *ENSECAG00000052043* | Protein coding | - | 20 | 51,685,275-51,699,694 |  |
| *GSTA4* | Protein coding | glutathione S-transferase alpha 4 | 20 | 51,710,054-51,725,846 |  |
| *CILK1* | Protein coding | ciliosis associated kinase 1 | 20 | 51,729,979-51,778,641 |  |
| *ENSECAG00000052794* | Protein coding | - | 20 | 51,764,344-51,787,487 |  |
| *FBXO9* | Protein coding | F-box protein 9 | 20 | 51,787,279-51,809,250 |  |
| *GCM1* | Protein coding | glial cells missing transcription factor 1 | 20 | 51,834,661-51,854,473 |  |
| *ENSECAG00000045553* | LncRNA | - | 20 | 51,872,077-51,900,613 |  |
| *ENSECAG00000039105* | LncRNA | - | 20 | 51,909,253-51,911,622 |  |
| *ENSECAG00000060298* | LncRNA | - | 20 | 51,906,412-51,921,291 |  |
| *ELOVL5* | Protein coding | ELOVL fatty acid elongase 5 | 20 | 51,920,493-51,963,377 |  |
| *ENSECAG00000048124* | LncRNA | - | 20 | 52,004,949-52,048,285 |  |
| *ENSECAG00000053866* | LncRNA | - | 20 | 52,006,033-52,048,323 |  |
| *ENSECAG00000046047* | LncRNA | - | 20 | 52,050,310-52,072,486 |  |
| *ENSECAG00000059441* | LncRNA | - | 20 | 52,077,518-52,081,538 |  |
| *GCLC* | Protein coding | glutamate-cysteine ligase catalytic subunit | 20 | 52,096,310-52,139,042 |  |
| *ENSECAG00000060270* | LncRNA | - | 20 | 52,140,483-52,143,448 |  |
| *ENSECAG00000047889* | LncRNA | - | 20 | 52,142,758-52,143,921 |  |
| *ENSECAG00000033927* | LncRNA | - | 20 | 52,143,621-52,216,884 |  |
| *ENSECAG00000050488* | LncRNA | - | 20 | 52,179,154-52,221,175 |  |
| *ENSECAG00000052085* | LncRNA | - | 20 | 52,186,296-52,190,256 |  |
| *ENSECAG00000056920* | LncRNA | - | 20 | 52,220,339-52,222,776 |  |
| *KLHL31* | Protein coding | kelch like family member 31 | 20 | 52,228,106-52,391,836 |  |
| *LRRC1* | Protein coding | leucine rich repeat containing 1 | 20 | 52,393,524-52,518,972 |  |
