# Supplementary material for: The genetics of gaits in Icelandic horses goes beyond DMRT3, with RELN and STAU2 identified as two new candidate genes
Source: Genet Sel Evol. 2023 Dec 11;55:89. doi: 10.1186/s12711-023-00863-6 (PMC10712087; doi:10.1186/s12711-023-00863-6)
Supplement: Supplementary file 9 — Additional file 9: Table S4. Number of horses within each group of horses with variable pacing ability. The number of horses with different haplotypes on ECA4 and ECA9 in groups of horses with variable pacing ability. [file 12711_2023_863_MOESM9_ESM.docx]

|  |  | **All horses** | **AA horses** | **5g AA horses (pace>5.0)** | **CA horses** | **4g AA horses (pace=5.0)** | **Pace racers** |
| --- | --- | --- | --- | --- | --- | --- | --- |
| **Haplotype on ECA 4** | **RR** | 102 | 93 | 78 | 9 | 12 | 3 |
|  | **Rr**^†^ | 205 | 189 | 138 | 15 | 44 | 7 |
|  | **rr** | 65 | 58 | 32 | 7 | 26 | 0 |
| **Haplotype on ECA 9** | **SS** | 305 | 276 | 217 | 28 | 52 | 7 |
|  | **Ss**^†^ | 62 | 59 | 29 | 3 | 27 | 3 |
|  | **ss** | 5 | 5 | 2 | 0 | 3 | 0 |

^†^The groups referred to as heterozygous individuals (Rr and Ss) also included horses possessing the rare haplotypes from the haplotype analyses (1% of the sample size with the rare haplotypes on ECA 4 and 2% of the sample size with the rare haplotypes on ECA 9).
